# Chronic low dose ^90^Sr contamination in *Lemna minor*: from transcriptional dynamics of epigenetic regulators to population level effects

**DOI:** 10.3389/fpls.2025.1605017

**Published:** 2025-06-26

**Authors:** Luca Boldrini, May Van Hees, Gustavo Turqueto Duarte, Robin Nauts, Jean Wannijn, Yelltrich Reymen, Brix De Rouck, Hilde Loots, Matteo Schiavinato, Henriette Selck, Nele Horemans

**Affiliations:** ^1^ Unit for Biosphere Impact Studies, Belgian Nuclear Research Centre (SCK CEN), Mol, Belgium; ^2^ Department of Science and Environment, Roskilde University, Roskilde, Denmark; ^3^ Centre for Environmental Sciences, University of Hasselt, Hasselt, Belgium; ^4^ Integrated Molecular Plant Physiology Research (IMPRES), University of Antwerp, Antwerpen, Belgium; ^5^ Wageningen University & Research, Wageningen, Netherlands

**Keywords:** ecotoxicology, plants, risk assessment, Chernobyl, ionizing radiation, abiotic stress, epigenetics, long-term exposure

## Abstract

The ecotoxicology model plant *Lemna minor* was exposed for 6 weeks to ^90^Sr, simulating the dose rates present in the Chernobyl Exclusion Zone (CEZ), in order to understand the effects of chronic low dose ionising radiation exposure. The data suggest that the plant may exhibit temporally variable acclimation responses that can be interpreted as early-, mid-, and long-term phases. Morphological changes included increased area and frond number, while molecular adjustments encompassed variations in pigment levels, glutathione metabolism, and expression modulation of telomerase-related and DNA methylation machinery genes. Physiological parameters and ^90^Sr uptake remained relatively stable, yet fluctuations indicate a continuous adjustment to the chronic stress, suggesting *L. minor’*s potential for phytoremediation. The interplay between transcriptional regulation of DNA methylation and the examined endpoints suggests a potential involvement of epigenetic mechanisms in *L. minor*’s acclimation to chronic low dose-rate ^90^Sr stress. This work provides knowledge on *L. minor*’s abiotic stress responses and contributes to our understanding of plant adaptation to low-level ionising radiation (IR). The findings contribute to the development of adverse outcome pathways (AOPs) for *L. minor* exposed to IR, improving environmental risk assessment approaches.

## 1 Introduction

Every living organism is constantly exposed to background levels of ionising radiation (IR) originating from both natural and anthropogenic sources (Ramachandran, 2011). The former, a constant and unavoidable aspect of life on Earth for every organism, retains biological relevance for the majority of individuals (UNSCEAR, 2000; Turqueto Duarte et al., 2023). The latter includes non-accidental radioactive releases such as those from mining, milling, and industrial processes, and also pertains to exceptional occurrences, such as the Chernobyl nuclear explosion in 1986.

Release of radioactive materials into the environment during radioactive fallouts and nuclear weapon testing (Machlis and Hanson, 2008), and catastrophic events like nuclear power plant accidents can cause substantial impact on biota (Turqueto Duarte et al., 2023). In particular, the Chernobyl accident led to enhanced environmental exposure to low levels of radionuclides in the surrounding area that will last for decades, inducing chronic long-term low-dose radiation stress to the biota. Nevertheless, the accident provided the scientific community with priceless chances to examine the long-term fate of radionuclides and their biological consequences at different levels of complexity (Sample and Irvine, 2011). Data from Chernobyl field studies and laboratory trials were compared, and they showed an intricate picture of radiosensitivity: wildlife in field conditions showed increased radiosensitivity than those tested in controlled environment, potentially due to the combined effects of radiation and other environmental stressors (Adam-Guillermin et al., 2018). Conversely, research on *Arabidopsis thaliana* plants inhabiting the “Chernobyl Exclusion Zone” (CEZ) indicated the development of radioresistance in subsequent generations, as evidenced by reduced sensitivity to new acute IR exposures (Podlutskii et al., 2022). Unquestionably, exposure time is one of the key characteristics that differentiates laboratory studies from field trials, and it can provide a partial explanation to such a discrepancy. While laboratory exposures are often limited in time and generally employ acute exposure setups, organisms in field conditions are typically exposed in a chronic, multigenerational fashion (Garnier-Laplace et al., 2013; Mishra et al., 2024). The relatively long-lived ^90^Sr radionuclide (28.79 years physical half-life) is a pure β-emitter which is still currently found in the highly polluted drainage basins and lakes of the CEZ (Igarashi et al., 2020). Although not essential for plant primary metabolism (Maathuis, 2009), Sr shares chemical similarities with the calcium (Ca) macronutrient (Chowdhury and Blust, 2011; Pecher, 1941). Apparently, plants can store Sr making use of the Ca uptake and transport systems (Van Hoeck et al., 2015b). As surface tissues receive the majority of β-particles energy, bioaccumulation resulting from acute exposures to this radionuclide is indeed reported to generate biological damage (Van Hoeck et al., 2015b). While in-depth analyses on the biological effects of ^90^Sr β-radiation in plants are still limited (Copplestone et al., 2010), evidence is mounting that it can induce multiple effects at different levels of biological complexity in both crops (Kodaira, 1964; Tensho et al., 1959) and non-crop plants (Duarte et al., 2019; Kamra, 1974).

In general, when IR interacts with plant cells, it induces a variety of direct and indirect effects (Alizadeh et al., 2015; Esnault et al., 2010). First, through direct energy deposition, IR induces DNA damage as a primary effect, which can manifest as DNA strand breaks and base damage (Duarte et al., 2023; Nakano et al., 2017). Furthermore, IR indirectly gives rise to oxidative stress through an excessive generation of reactive oxygen species (ROS), primarily due to water radiolysis (Mishra et al., 2024). Additional sources include activation of ROS-producing enzymes, such as the NADPH oxidase/respiratory burst oxidase homolog (RBOH), whose gene upregulation has been observed upon IR exposure (Song et al., 2024; Vanhoudt et al., 2008). However, ROS are also a common byproduct of plant’s photosynthetic metabolism (García-Caparrós et al., 2021), and serve as signalling molecules when present at low concentrations (Ullah Khan and Wilson, 1995). When their production exceeds the plant’s scavenge capacity, these molecules impact the homeostasis of the system, and are therefore perceived as redox stressors by the cell (Farooq et al., 2019). Consequently, radiostrontium-induced stress in plants is reported to influence the ROS scavenging system, affecting both non-enzymatic (e.g., GSH) and enzymatic components (e.g. superoxide dismutase (SOD), catalase (CAT), and peroxidases (PX)) (Ali et al., 2022; Burger and Lichtscheidl, 2019; Esnault et al., 2010; Van Hoeck et al., 2015b; Zaka et al., 2002). In addition to components directly involved in ROS scavenging, IR stress responses can involve ROS-producing or -consuming enzymes like guaiacol peroxidase (GPOD) (van de Walle et al., 2016; Vanhoudt et al., 2014; Venugopal et al., 2016). This peroxidase enzyme located in cytosol, vacuole, cell wall, apoplast and extracellular medium, plays an important role in plant development (Ghamsari et al., 2007) and influences redox balance during stress by consuming hydrogen peroxide (H_2_O_2_) (Cho and Seo, 2005; Šimonovičová et al., 2004). Previous research by Van Hoeck et al. on the effects of chronic gamma radiation on *Lemna minor* demonstrated that GPOD showed increased activity during chronic IR stress exposure (Van Hoeck et al., 2015c).

To cope with chronic IR-induced redox stress, plants rely, among others, on dose-dependent transcriptional (Van Hoeck et al., 2017) and epigenetic regulations (Laanen et al., 2021), the latter with a primary focus on changes in DNA methylation patterns (Bondarenko et al., 2023; Horemans et al., 2018; Kovalchuk et al., 2003; Laanen et al., 2023, 2021; Volkova et al., 2018). Nevertheless, the input of epigenetic changes on responses to IR exposure remains widely unexplored. In general, DNA methylation has two main functions: first, it orchestrates organismal growth and development by fine tuning gene expression; and secondly, it gives plants a powerful and fast responsive molecular tool for controlling biotic and abiotic stress responses (Kumar and Mohapatra, 2021). Evidence suggests that this might be a relevant evolutionary mechanism for plants with a clonal reproductive mechanism (Douhovnikoff and Dodd, 2015). Unlike sexually reproducing plants, clonal plants bypass the epigenetic resetting process of meiosis (Paszkowski and Grossniklaus, 2011; Tricker, 2015), allowing for the preservation of environmentally induced epigenetic modifications across generations.

While the induction of epigenetic changes promoted by chronic β-radiation is only beginning to be uncovered (Georgieva et al., 2017; Horemans et al., 2018), external γ radiation has been shown to induce changes in epigenetic regulatory targets in various plant species (Laanen et al., 2023, 2021; Lee et al., 2020). In addition, evidence from both lab and field studies highlighted the relevance of these epigenetic changes in plants exposed to chronic, low-dose IR (Horemans et al., 2019). For instance, Volkova et al. observed alterations in transposable elements, chaperones, and histones (components of the epigenetic regulation system, influencing chromatin structure and gene expression) in herbaceous species grown in CEZ field conditions (Volkova et al., 2021). Similarly, Turqueto Duarte et al. found that transposon activity in *Pinus sylvestris* of the CEZ inversely correlated with chronic radiation levels (Duarte et al., 2019), while Bondarenko et al. reported varying DNA methylation patterns in *P. sylvestris* from the CEZ and Fukushima (Bondarenko et al., 2023). Although these studies suggest a clear link between radiation exposure and epigenetic changes in various plants, comparable research on *L. minor* is still relatively scarce and usually focuses on more standard endpoints. For instance, studies on *L. minor* exposed to chronic low-dose γ radiation have reported measurable effects at multiple levels of biological organization, including phenotypic differences such as decreased frond number (Ivanishvili et al., 2016) and altered gene expression (Van Hoeck et al., 2017).

In order to understand the consequences of multigenerational chronic effects caused by the exposure to long-lasting radionuclides, the present work focused on strontium-90 (^90^Sr), which due to its physical half-life will remain present in the CEZ for many decades to come. To this end, the clonally reproducing and ecotoxicology monocot freshwater model *L. minor* was grown for six weeks in ^90^Sr-contaminated medium. *L*. *minor* is an asexual, fast-reproducing plant with an average generation time of 2.5 days (Gonzales et al., 2023; OECD, 2006), making it an ideal model organism for long-term chronic exposure studies. Ecological realism was simulated by reproducing Chernobyl-like activities (*e*.*g*. 40 Bq/L) (Shevchenko et al., 2001) with the following aims: 1. Mechanistically understanding plants’ responses to chronic IR-induced stress at different levels of biological organization; 2. Examining *L. minor* chronic IR responses over a longer exposure period (6 weeks), compared to the commonly used chronic 1-week test. This extended exposure better simulates field conditions and provides deeper insights into chronic responses; 3. Suggesting improvements for the environmental risk assessment (ERA) approaches currently in use.

Our work highlights the importance of chronic and low dose IR stress in ERA, as well as its complex relationship to plant epigenetics and biochemical stress responsive mechanisms. By deepening our understanding of the aforementioned molecular mechanisms, this research provides data that can serve to ameliorate environmental risk assessment methods and, hence, are relevant to the management of IR-related ecological challenges.

## 2 Materials and methods

The **Supplementary Material** (**Supplementary Methods**) provides detailed descriptions of ^90^Sr uptake, dose rate calculations using the ERICA tool, DNA extraction, qRT-PCR for gene expression and telomere length analyses, and measurements of physiological, biochemical, and molecular endpoints such as photosynthetic activity, pigment and glutathione levels, guaiacol peroxidase activity, and macromolecular contents (proteins, sugars, and starch).

### 2.1 Plant material and contamination setup

*L*. *minor* (serial number 1007 and ID number 5500) clones were employed in the experiments. Briefly, it was carried out as follows: plants were cultured in sterile 250 mL glass flasks filled with 100 mL of 1/10 strength Hoagland solution (1 mM KNO_3_; 0.3 mM Ca(NO_3_)2·4H_2_O; 0.2 mM MgSO_4_·7H_2_O; 0.1 mM NH_4_H_2_PO_4_; 1.6 μM FeSO_4_.7H_2_O; 0.8 μM EDTA diNa-salt 2H_2_O; 4.6 μM H_3_BO_3_; 0.9 μM MnCl_2_·4H_2_O; 0.03 μM CuSO_4_·5H_2_O; 0.06 μM H_2_MoO_4_; 0.08 μM ZnSO_4_·7H_2_O; pH 5.6) (Van Hoeck et al., 2017). Plant culture prior to the experiment was conducted under sterile conditions, with plants positioned on the surface of the medium exclusively using sterilized inoculation loops under a laminar flow. Then, the experiment was maintained under semi-sterile conditions using transparent sterile round Petri plates placed on top of the pots. This was done to minimize potential contamination and to further reduce evaporation and limit particle deposition while allowing air exchange. The growth conditions were set to 16h day and 8h night photoperiod (150 μmol m^-2^ s^-1^ at the leaf level), day/night temperatures of 22°C/18°C in a controlled climate chamber (Microclima MC1000E, Snijders Scientific B.V., Tilburg, The Netherlands).

Upfront of each experiment, the plants were initially pre-cultured for two weeks in sterilised transparent pots (Nalgene^®^, Thermo Fisher Scientific) in 50 mL of 1/10 strength Hoagland solution, following the experiment phase under similar conditions. It was tested beforehand in a pilot experiment that 50 mL was an appropriate volume for the growth of 7 plants over 7 days, with no growth limitation observed (**Supplementary Figure 1**). The pre-culture process started with 7 plants, and at the beginning of every following week 7 plants were sub-cultured to new pots with fresh Hoagland medium. For each experimental condition, 15 pots were used as biological replicates.

At the start of the 6-weeks contamination experiments, a ^90^Sr source (SrCl_2_, 485895 kBq/L) was diluted in demineralized water and filtered (0.2 µm), added to the respective media, and the pH was adjusted to 5.6 with NaOH. Four different activity concentrations were prepared in 1/10 strength Hoagland medium: β0 = 0 Bq/L (control), β1 = 40 Bq/L, β2 = 400 Bq/L and β3 = 4000 Bq/L. For each time point, 60 sterile 250 mL clear Nalgene^®^ pots (15 for the control and 15 per each contaminated condition) were prepared with 50 mL of fresh sterile 1/10 Hoagland medium solution per week. The start of each week (Day 0, Week N_1-6_) was marked by the transfer of 7 mature plants with 3–4 fronds from the previous week’s culture to new pots, following the respective contamination condition. For the first week, all plants used for every condition came from the pre-culture. Transfer of plants was carried out at the same time during every week. Plants’ growth was followed for the whole duration of the experiment (i.e. 6 weeks). In order to automatize measurements of area growth rates, a 1 cm^2^ sterilized red plastic square reference was placed floating on top of the medium. To prevent excessive evaporation of the medium while yet allowing necessary light transmission and environment exchange, each pot was covered with a clear, sterile Petri dish. To guarantee appropriate homogeneity for all conditions and replicates, the pots were randomly distributed inside the climate chamber. At day 7 of each week, all plants but those transferred to the subsequent week were harvested and immediately analysed (e.g. photosynthetic parameters measurements, pigment content evaluations) or stored at -80°C for future analytical purposes.

Top-view photographs were taken from the same distance, employing the same light conditions and camera settings on days 0, 2, 4 and 7 of each week to track frond growth over the course of the experiment. Images were processed using the open source software EasyLeafArea version 2.0 (Easlon and Bloom, 2014) and ImageJ version 1.8.0 (Schneider et al., 2012), through which the total surface area and number of the fronds was obtained. Subsequently, following the OECD guidelines for toxicity testing of the genus *Lemna* (Test Method No. 221: *Lemna* sp), average specific growth rates (ASGR) were calculated for the frond number and area, according to the following formula:


$$\mu_{i-j}=\frac{\ln(N_{j})-\ln(N_{i})}{t}$$

where:

- $\mu_{i-j}$ : average specific growth rate from time i to j

- $N_{i}$ : measurement variable in the test or control vessel at time i

- $N_{j}$ : measurement variable in the test or control vessel at time j

- *t*: time period from *i* to *j*

- *i*: day 0 of each week

- *j*: day 7 of each week

### 2.2 Statistical analysis

Normality of the data was evaluated through the Shapiro-Wilk test using R software (R version 4.3.3) (R Core Team, 2023). For data sets that exhibited normal distribution, a two-way ANOVA was performed, followed by *post hoc* Tukey’s multiple comparison tests to identify significant differences between groups, all carried out using GraphPad software (GraphPad prism version 9.4.1). When normality was not met, the non-parametric Kruskal-Wallis test was applied. In such cases, *post hoc* comparisons were conducted using Dunn’s test with Bonferroni correction to account for multiple comparisons, also carried out through the GraphPad software. For gene expression data, the Wilcoxon Rank-Sum Test was used, conducted using R software (R version 4.3.3).

## 3 Results

### 3.1 ^90^Sr uptake and dosimetry

*L*. *minor* plants were grown for up to 6-weeks under varying activity concentrations of ^90^Sr (40 Bq/L, 400 Bq/L, and 4000 Bq/L) to gain insights into multigenerational dose- and time-dependent effects of chronic β-radiation exposure. The calculated average total dose rates were 0.82 ± 0.07 µGy•h^-1^ for β1, 7.69 ± 0.98 µGy•h^-1^ for β2 and 72.67 ± 6.90 µGy•h^-1^ for β3 and reflect the tenfold differences among β1, β2 and β3 activity concentrations employed in the experiment (**Table 1**). The total dose rate is the sum of the calculated internal and the external dose rate. Overall, internal absorbed dose rates were one order of magnitude greater than external.

**Table 1:** Dosimetric parameters of the 6-week ^90^Sr exposure experiment on L. minor plants.

Timepoint	Activity concentration		Dosimetric parameters					
	Nominal [Bq L^-1^]	Measured [Bq L^-1^]	Internal dose rate [µGy h^-1^]		External dose rate [µGy h^-1^]		Total dose rate [µGy h^-1^]	
			mean	SD	mean	SD	mean	SD
week 1	40	42	0.83	0.049	0.11	0.0067	0.83	0.05
week 2		42	0.88	0.017	0.12	0.0023	0.89	0.017
week 3		42	0.69	0.069	0.095	0.0094	0.7	0.069
week 4		41	0.75	0.048	0.1	0.0065	0.76	0.048
week 5		43	0.83	0.17	0.11	0.024	0.84	0.17
week 6		40	0.86	0.1	0.12	0.013	0.87	0.099
week 1	400	400	7.5	3.4	1	0.47	7.6	3.4
week 2		400	6	0.82	0.82	0.11	6.1	0.83
week 3		400	7.4	1.1	1	0.15	7.4	1.1
week 4		400	8	0.29	1.1	0.039	8.1	0.29
week 5		400	6.9	0.64	0.94	0.088	6.9	0.65
week 6		400	9.2	0.57	1.3	0.078	9.3	0.58
week 1	4000	4000	74	5.5	10	0.75	75	5.6
week 2		4400	73	0.74	10	0.1	74	0.75
week 3		4000	71	6.1	9.6	0.83	71	6.2
week 4		4000	58	15	7.9	2	59	15
week 5		3900	81	9.2	11	1.3	82	9.3
week 6		4000	75	4	10	0.55	75	4.1

Four different activity levels of IR from a ^90^Sr source were tested (0 Bq/L, 40 Bq/L, 400 Bq/L, and 4000 Bq/L) and samples were collected on the 7th day of each week. The table provides details on nominal and measured activity concentrations of ^90^Sr dosimetry parameters encompassing internal, external, and total dose rates. All provided data points are presented as mean and standard deviation, with three biological replicates. Control samples (0 Bq/L) were not measured as values were below the detection threshold.

To assess biomass dynamics upon ^90^Sr exposure, both fresh (FW) and dry weight (DW) were measured (**Table 2**). Statistically significant differences were observed in plants exposed to the intermediate (β2) and highest (β3) activity concentrations during week 3, 4 and 6, with the contaminated plants exhibiting higher values than the control plants. In contrast, concentration ratios of ^90^Sr expressed on both fresh and dry weight and dosimetric parameters (internal, external and total dose rates) remained stable throughout the full experiment.

**Table 2:** Biomass and Sr uptake of L. minor plants over 6-week ^90^Sr exposure.

Timepoint	Activity concentration	Biomass				Uptake parameters					
	Nominal [Bq L^-1^]	FW [mg]		DW [mg]		Uptake [Bq/g FW]		FW Conc. Ratio [Bq g^-1^ FW / Bq mL^-1^]		DW Conc. Ratio [Bq g^-1^ FW / Bq mL^-1^]	
		mean	SD	mean	SD	mean	SD	mean	SD	mean	SD
week 1	0	0.14	0.0096	0.0093	0.0015						
week 2		0.23	0.0015	0.013	0.0006						
week 3		0.28	0.039	0.011	0.0008						
week 4		0.23	0.024	0.011	0.0007						
week 5		0.20	0.0085	0.013	0.0012						
week 6		0.24	0.014	0.015	0.0010						
week 1	40	0.15	0.023	0.0087	0.0015	1.4	0.082	33	2	590	50
week 2		0.28	0.006	0.013	0.00301	1.5	0.028	35	0.67	814	207
week 3		0.27	0.017	0.013	0.0012	1.2	0.11	28	2.7	560	31
week 4		0.28	0.0075	0.0092	0.0061	1.3	0.08	30	1.9	701	38
week 5		0.3	0.075	0.013	0.0012	1.4	0.29	32	6.8	720	48
week 6		0.3	0.036	0.016	0.00052	1.5	0.15	38	3.9	730	56
week 1	400	0.23	0.13	0.01	0.0012	13	5.7	32	14	460	380
week 2		0.3	0.043	0.015	0.0015	10	1.4	25	3.5	490	44
week 3		0.47	0.16	0.025 **	0.0094	12	1.9	31	4.7	590	89
week 4		0.25	0.032	0.012	0.0019	13	0.48	34	1.2	710	36
week 5		0.26	0.013	0.013	0.00083	12	1.1	29	2.7	570	12
week 6		0.28	0.0069	0.016	0.00029	15	0.96	39	2.4	680	32
week 1	4000	0.16	0.0015	0.0077	0.00058	120	9.2	31	2.3	650	94
week 2		0.33	0.02	0.015	0.00058	120	1.2	28	0.28	590	11
week 3		0.25	0.037	0.013	0.00061	120	10	30	2.6	580	13
week 4		0.39 *	0.098	0.015	0.0003	97	25	24	6.2	620	8.1
week 5		0.29	0.036	0.014	0.0004	140	15	35	4	710	27
week 6		0.35 *	0.027	0.017 *	0.00068	130	6.8	32	1.7	660	10

Four different activity levels of IR from a ^90^Sr source were tested (0 Bq/L, 40 Bq/L, 400 Bq/L, and 4000 Bq/L) and samples were collected on the 7th day of each week. The biomass measurements are presented as fresh weight (FW) and dry weight (DW), and the uptake parameters expressed as concentration ratios. All provided data points are represented as mean and standard deviation, with three biological replicates. Statistically significant differences from control plants within the same week (Kruskal-Wallis and Dunn’s test for *post-hoc* comparisons) are indicated by an asterisk [significance levels: p-value < 0.05 (*), p-value < 0.01 (**), p-value < 0.001 (***), p-value < 0.0001 (****)]. Control samples (0 Bq/L) were not measured as values were below the detection threshold.

To assess the impact at the morphological level of 4 different ^90^Sr dose rates, frond area and number of *L*. *minor* plants were measured over a 6-week period. Measurements were carried out on days 0 (culture start), 2, 4, and 7 of each week (**Supplementary Figures 2**, **3**).

**Figure 1:**
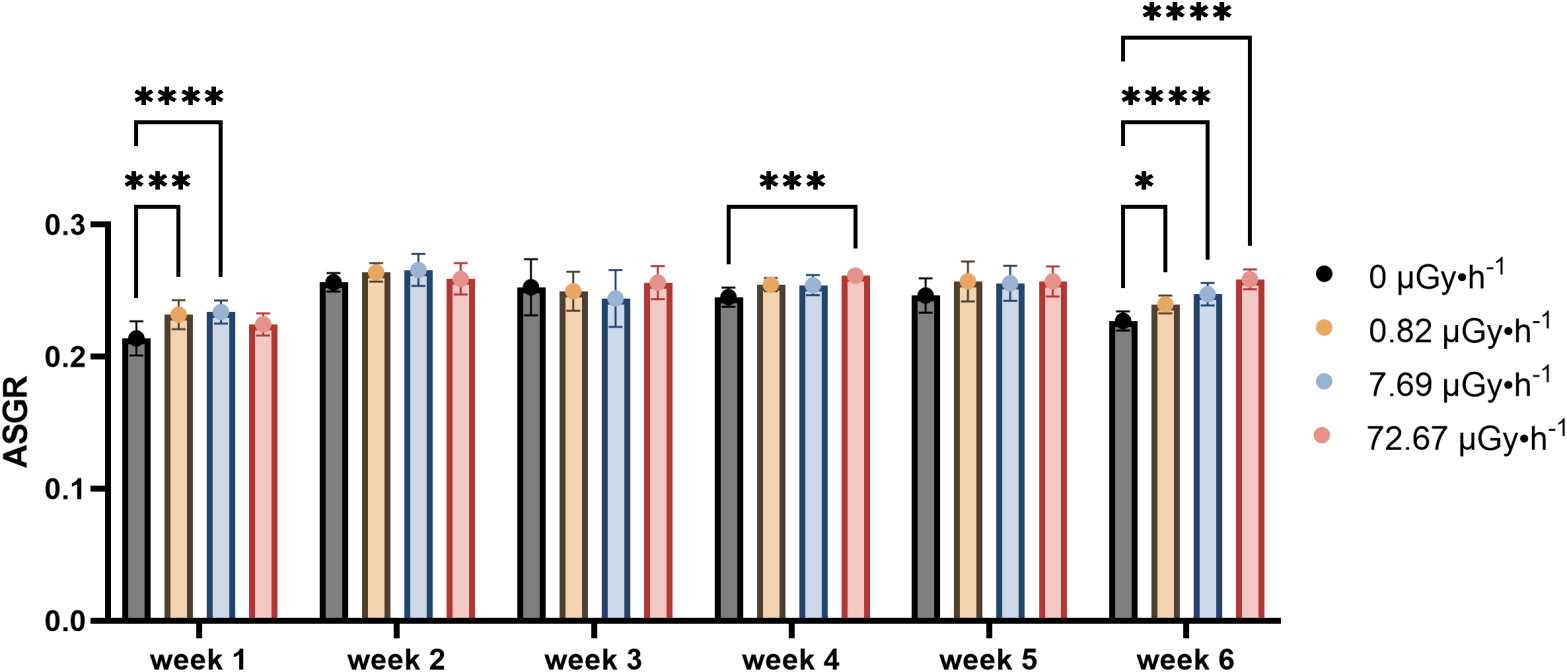
Average Specific Growth Rate (ASGR) of frond areas across weeks, representing the increase in frond areas on the 7^th^ day in comparison to day 0 of each week. Data are presented as mean ± SD (n=15). Plants were cultured in growth medium and exposed to different activity concentrations of IR from a ^90^Sr source: 0 (control), 0.82 (β1), 7.69 (β2) and 72.67 (β3) µGy•h^-1^. Within week statistical significance is represented by an asterisk (p-value < 0.05 (*), p-value < 0.01 (**), p-value < 0.001 (***), p-value < 0.0001 (****); two-way ANOVA).

The average specific growth rate (ASGR, calculated for both frond area and frond number as described in the OECD guideline 226) provides a quantification for comparisons between different treatments. A statistically significant difference in area ASGR was observed between the control group and the β1 and β2 groups during week 1 (**Figure 1**), which disappeared during weeks 2 and 3. Statistically significant difference in frond area was again observed in the highest dose rate group (β3) of week 4 and 6, which may reflect a cumulative or delayed effect of ^90^Sr radiation exposure. While no significant difference was observed in week 5, it is possible that the higher heterogeneity of the plants in that week hid a potential difference from control. Following the same pattern observed earlier, the group β3 displayed the greatest difference in frond area from the control group during week 6.

**Figure 2:**
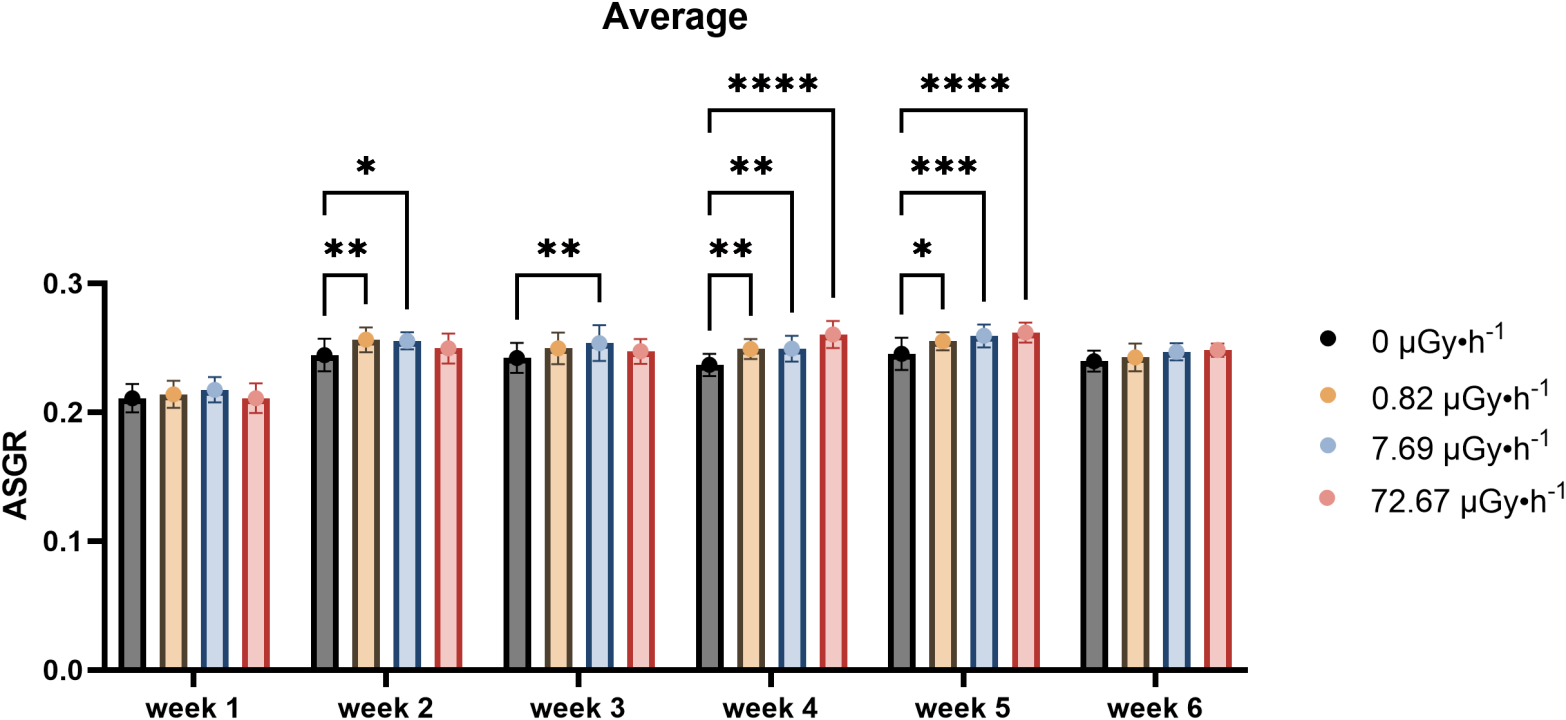
Average Specific Growth Rate (ASGR) of frond number across weeks, representing the increase in frond number on the 7^th^ day in comparison to day 0 of each week. Data are presented as mean ± SD (n=15). Plants were cultured in growth medium and exposed to different activity concentrations of IR from a ^90^Sr source: 0 (control), 0.82 (β1), 7.69 (β2) and 72.67 (β3) µGy•h^-1^. Within week statistical significance is represented by an asterisk (p-value < 0.05 (*), p-value < 0.01 (**), p-value < 0.001 (***), p-value < 0.0001 (****); two-way ANOVA).

The analysis of the number of fronds revealed a within-week specific increase under contaminated conditions, starting in the second week and persisting until the fifth week (**Figure 2**). Frond number and frond area showed partially overlapping dynamics, although the timing of statistically significant differences did not coincide exactly. This suggests that while both traits reflect growth responses to ^90^Sr exposure, they may do so with differing sensitivities or temporal patterns. In particular, during the first two weeks, a hormetic-like effect could be detected for the two lowest dose rates (β1 and β2), with slightly reduced growth observed under the highest dose rate (β3). In weeks 4 and 5, this hormetic-like response became more pronounced, including the β3 condition, in a similar way to what happened in the weeks 4–6 to area values. Specifically, increased values were observed for β1 and β2 conditions in week 2, for β2 in week 3, and for all non-control conditions (β1, β2, and β3) in weeks 4 and 5, with week 4 showing comparable changes to the ones seen for area ASGR for the highest condition.

### 3.2 Telomere length estimation

Telomeres, the nucleoprotein structures protecting chromosome ends, play a critical role in maintaining genome stability (Blackbum, 1991; McKnight et al., 2002). Although IR exposure is reported to induce genome instability in plants (Duarte et al., 2023; Kim et al., 2019; Yokota et al., 2010), not much is understood about the influence of IR on telomere length, despite the abundant data documenting this link in mammals (Bains et al., 2019; Biswas et al., 2024; Goytisolo et al., 2000; Ilyenko et al., 2011; Movahedi et al., 2019). To address this gap, relative telomere length measurements across all experimental conditions and time points were carried out. Telomere length remained stable across all experimental conditions and time points (**Supplementary Figure 4**). Statistical analysis revealed no significant differences in telomere length among the control group and the contaminated groups. This suggests that although β-radiation does not alter the overall length of telomeres, it remains to be investigated whether it is due to damage avoidance mechanisms or if a possible reduction could be compensated by increased damage repair.

### 3.3 Photosynthetic parameters

The electron transport rate (ETR) represents one of the key photosynthetic endpoints commonly assessed during the analysis of the effects of IR on photosynthetic organisms. For instance, acute γ exposure is reported to cause a decrease in ETR in the algae *Chlamydomonas reinhardtii* (Gomes et al., 2017) and the aquatic plant *Zizania latifolia* (Fan et al., 2014), whereas chronic IR has been shown to enhance ETR in *Arabidopsis thaliana* subjected to low dose rates of α (Biermans et al., 2015) and γ radiation (Vanhoudt et al., 2014). Therefore, to gain insights on *L. minor*’s response during chronic low dose exposure over multiple generations, the ETR in function of the photosynthetic active radiation (PAR) was assessed over a 6-week exposure period (**Supplementary Figure 5**). Photosynthetic efficiency in function of the light intensity appears to remain unaltered under long-term ^90^Sr contamination in comparison to the control (**Supplementary Figure 5**), with the exception of week 2 (**Supplementary Figure 5**), where there is an observed decline ETR across all the conditions.

**Figure 3:**
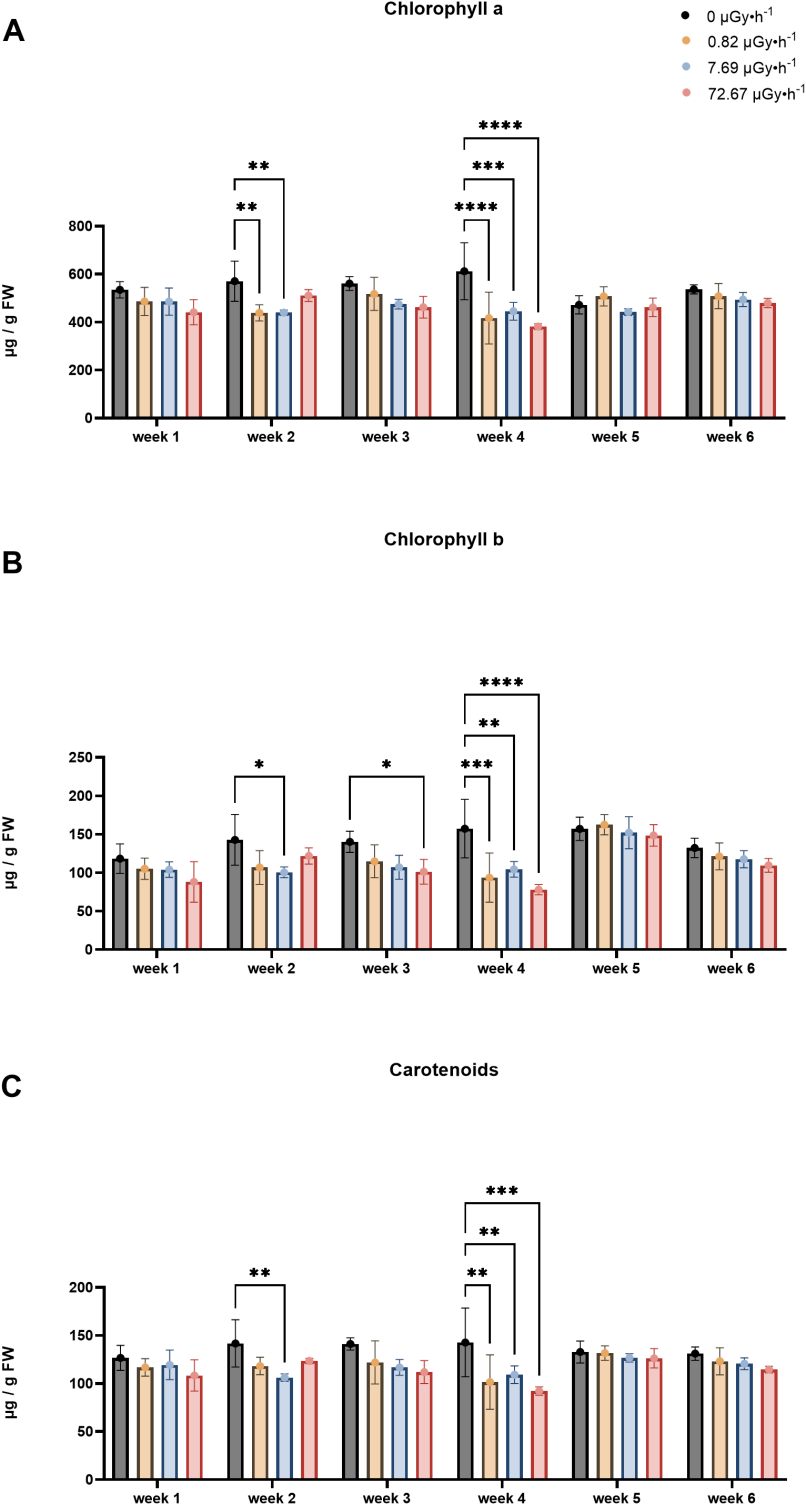
Pigment content fluctuations: **(A)** Chlorophyll a content; **(B)** Chlorophyll b content; **(C)** Carotenoids content. Data are presented as mean ± SD (n=3). Plants were cultured in growth medium and exposed to different activity concentrations of IR from a ^90^Sr source: 0 (control), 0.82 (β1), 7.69 (β2) and 72.67 (β3) µGy•h^-1^. Within week statistical significance is represented by an asterisk (p-value < 0.05 (*), p-value < 0.01 (**), p-value < 0.001 (***), p-value < 0.0001 (****); two-way ANOVA). µGy, microgray; h, hour.

IR reportedly affects the concentration of photoactive pigment in plants (Kim et al., 2004; Sengupta et al., 2013). Thus, *L. minor*’s chlorophyll a, b, and carotenoid levels were measured to gain information about chronic IR responses at the physiological and biochemical level (**Figures 3A–C**). For chlorophyll a, a generally lower content was displayed in plants exposed to ^90^Sr IR during all sampling weeks (**Figure 3A**). The reduction was statistically significant for groups β1 and β2 in week 2 (p < 0.01) and week 4 (p < 0.0002), and for group β3 in week 4 (p < 0.0001) (**Figure 3A**). Despite the lack of statistically significant variations in weeks 1, 3, 5, and 6, the detected trends suggested a potential involvement of chronic IR exposure in chlorophyll a levels.

The results for chlorophyll b (**Figure 3B**) indicated a trend of decreased content induced by chronic low dose exposure over multiple generations, observed across all time points, which was statistically significant for weeks 2, 3, and 4. Chlorophyll b is mostly present in the antennas rather than in photoactive cores, therefore the chlorophyll a/b ratio (Chl a/b) gives an estimate of the proportion of molecules bound by light-harvesting complexes (Walters, 2005). This additional parameter is a common endpoint used to monitor acclimation in plants and was also analyzed in this experiment. Here, acclimation is intended as a short-term, reversible physiological adjustment to environmental changes, distinct from adaptation, which involves long-term, heritable genotypic and phenotypic changes that persist beyond an individual’s lifetime (Collier et al., 2019; Kumarathunge et al., 2019; Mitra et al., 2021; Morgan-Kiss et al., 2006). In the context of this work, such acclimation responses may reflect the plant’s ability to retain function under mild but persistent stress conditions, i.e. a form of eustress (Van Hoeck et al., 2017). To this end, in week 1 and 4, significant increases in Chl a/b were observed only in the β3 groups (**Supplementary Figure 6**), which might indicate a high-dose threshold for initiating photosynthetic acclimation towards chronic IR in *L*. *minor*. This finding could also provide information on the steric restructuring of the photosynthetic apparatus in response to chronic low dose exposure over multiple generations, as a high Chl a/b ratio indicates acclimation to stress conditions, possibly involving reduced antennae size to avoid damage from excessive harvested photons (Walters, 2005).

Regarding the carotenoids (**Figure 3C**), β2 values were significantly lower than the control group (p = 0.005) in week 2. Overall, a consistent trend was observed for all the weeks, with the control group maintaining highest levels and the contaminated groups showing lower levels, as seen for chlorophyll a and b. This trend persisted and became statistically significant for all conditions in week 4, in the same way as reported for chlorophyll a and b (**Figures 3A–C**).

### 3.4 Total macromolecule content

It is reported that shifts of macromolecule levels are linked to stress responses in plants (Dubey and Singh, 1999; Gill et al., 2003; Huang et al., 2013; Pagliuso et al., 2018; Strand et al., 1999; Van Dyck et al., 2024). Therefore, total protein, sugar and starch content were measured to gain understanding about *L. minor*’s physiological state upon ^90^Sr stress. These measurements have been carried out on control, β2 and β3 plants.

**Figure 4:**
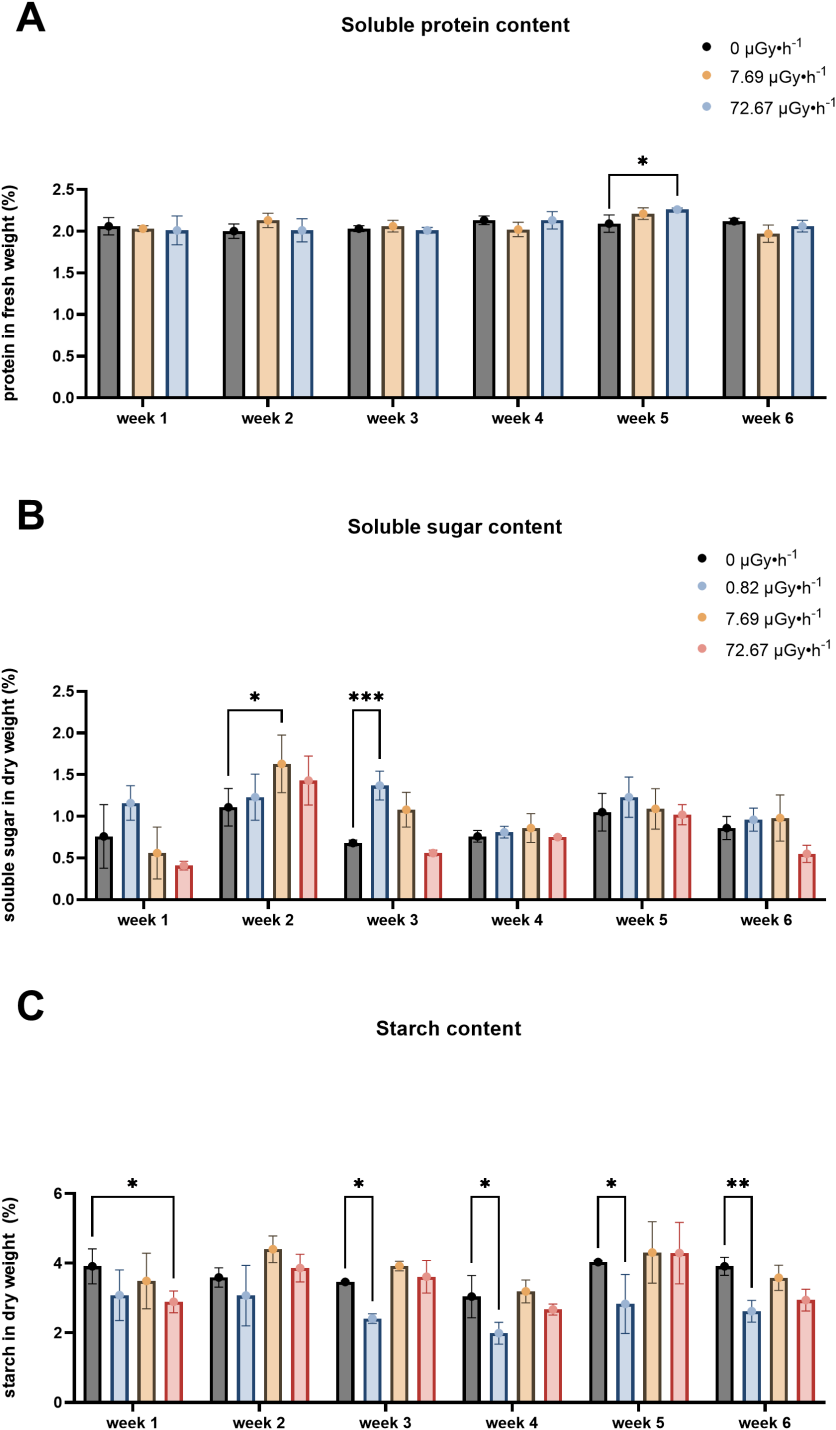
Macromolecular content fluctuations: **(A)** Soluble protein content, **(B)** Soluble sugar content, **(C)** Starch content. Data are presented as mean ± SD (n=3). Plants were cultured in growth medium and exposed to different activity concentrations of IR from a ^90^Sr source: 0 (control), 7.69 (β2) and 72.67 (β3) µGy•h^-1^ for the protein content analysis and 0, (control), 0.82 (β1), 7.69 (β2) and 72.67 (β3) µGy•h^-1^ for the soluble sugar and starch content analyses. Within week statistical significance is represented by an asterisk (p-value < 0.05 (*), p-value < 0.01 (**), p-value < 0.001 (***), p-value < 0.0001 (****); two-way ANOVA). µGy, microgray; h, hour.

While protein content is associated with stress responses in plants, there is no consensus on whether stress enhances or decreases its levels (Abdul Qados, 2011; Campbell et al., 1981; Doganlar et al., 2010; Gulen and Eris, 2004; Mohammadkhani and Heidari, 2008). In this study, protein content remained relatively stable throughout 6 weeks, with the exception of a slight increase observed in β3 samples during week 5 (**Figure 4A**). Considering the modest magnitude of this increase, it is plausible that this variation partly falls within the range of experimental variability.

Soluble sugar remained largely unchanged, except for increases observed in β2 plants during week 2 and in in β1 in week 3 (**Figure 4B**). This elevation might represent a stress acclimation mechanism which could be linked to photosynthetic performance (Boriboonkaset et al., 2013; Masoudi et al., 2011; Pociecha et al., 2010) or osmotic adjustment (Djanaguiraman et al., 2006; Dubey and Singh, 1999; Watanabe et al., 2000).

Starch accumulation (**Figure 4C**) is reported to increase in *L. minor* under stress conditions and it has been correlated to lower growth rates (Sree and Appenroth, 2014; Van Dyck et al., 2021). Interestingly, chronic exposure to ^90^Sr did not lead to higher starch levels, which were in fact reduced in β3 plants during weeks 1 and in β1 in weeks 3-6. With the exception of β1 plants, the overall stability of the three evaluated macromolecule levels (total protein, total sugars and starch) over the weeks suggested that *L*. *minor* effectively adjusted its metabolism to the presence of this radionuclide, which at the tested concentrations seemed to trigger low stress response.

### 3.5 Antioxidants

**Figure 5:**
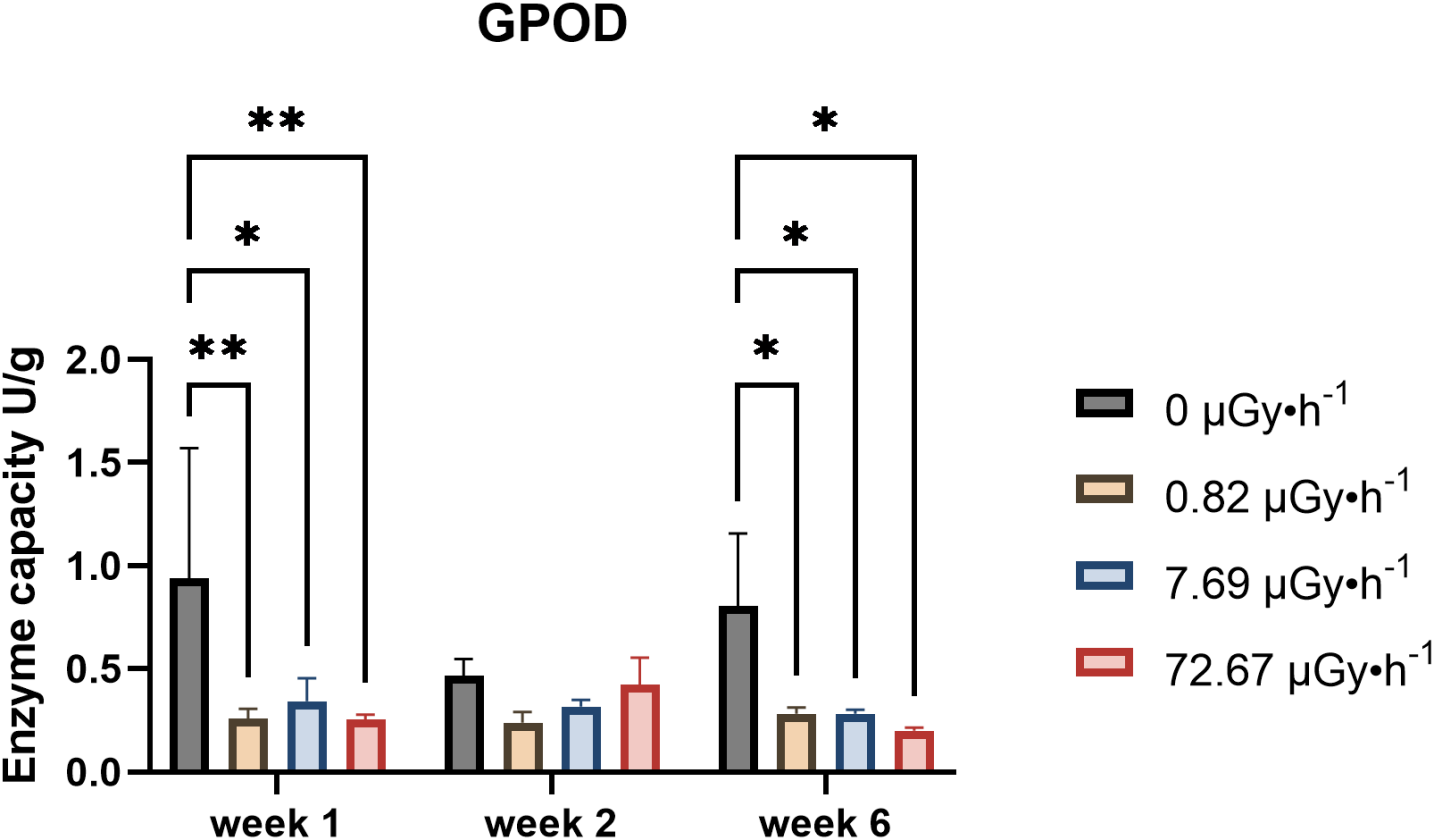
GPOD capacity fluctuations in week 1, 2 and 6. Data are presented as mean ± SD (n=3). Plants were cultured in growth medium and exposed to different activity concentrations of IR from a ^90^Sr source: 0 (control), 0.82 (β1), 7.69 (β2) and 72.67 (β3) µGy•h^-1^. Statistical significance is represented by an asterisk (p-value < 0.05 (*), p-value < 0.01 (**), p-value < 0.001 (***), p-value < 0.0001 (****); two-way ANOVA). µGy, microgray; h, hour.

Glutathione levels, both in their reduced and oxidized forms (GSH and GSSG, respectively), and peroxidases are often employed as antioxidative defence biomarkers. Reduced antioxidants and peroxidase enzymes can mitigate the effects of excess reactive oxygen species generated under stress, including IR exposure (Oztetik, 2015; Riley, 1994). Our investigation into glutathione content and its redox state revealed, however, that there was very little difference comparing control to exposed plants (**Supplementary Figure 7**). **Figure 5** represents the variation of guaiacol peroxidase (GPOD) in weeks 1, 2 and 6 (other weeks were not measured).

The results of GPOD activity showed, with the exception of week 2, a decreasing trend for all the conditions analysed which was statistically significant both in week 1 and 6. Overall, the data underlined time and dose-rate dependence of *L*. *minor*’s GPOD activity upon ^90^Sr contamination. Moreover, it is noteworthy that these patterns align with those observed for area ASGR. This correlation may be attributed to peroxidase’s role in cell loosening (Liszkay et al., 2003). Reduced GPOD activity could indicate decreased cell wall stiffening, potentially leading to more elongation growth and increased surface. While direct ROS quantification could offer additional insight, we strategically prioritized endpoints more central to our study objectives, including ^90^Sr uptake and gene expression related to antioxidant responses, given the limited biomass typical of *L. minor*. Future studies may build on this work by incorporating direct ROS measurements to further deepen understanding.

### 3.6 Gene expression changes suggest potential involvement of stress-related epigenetic mechanisms

**Figure 6:**
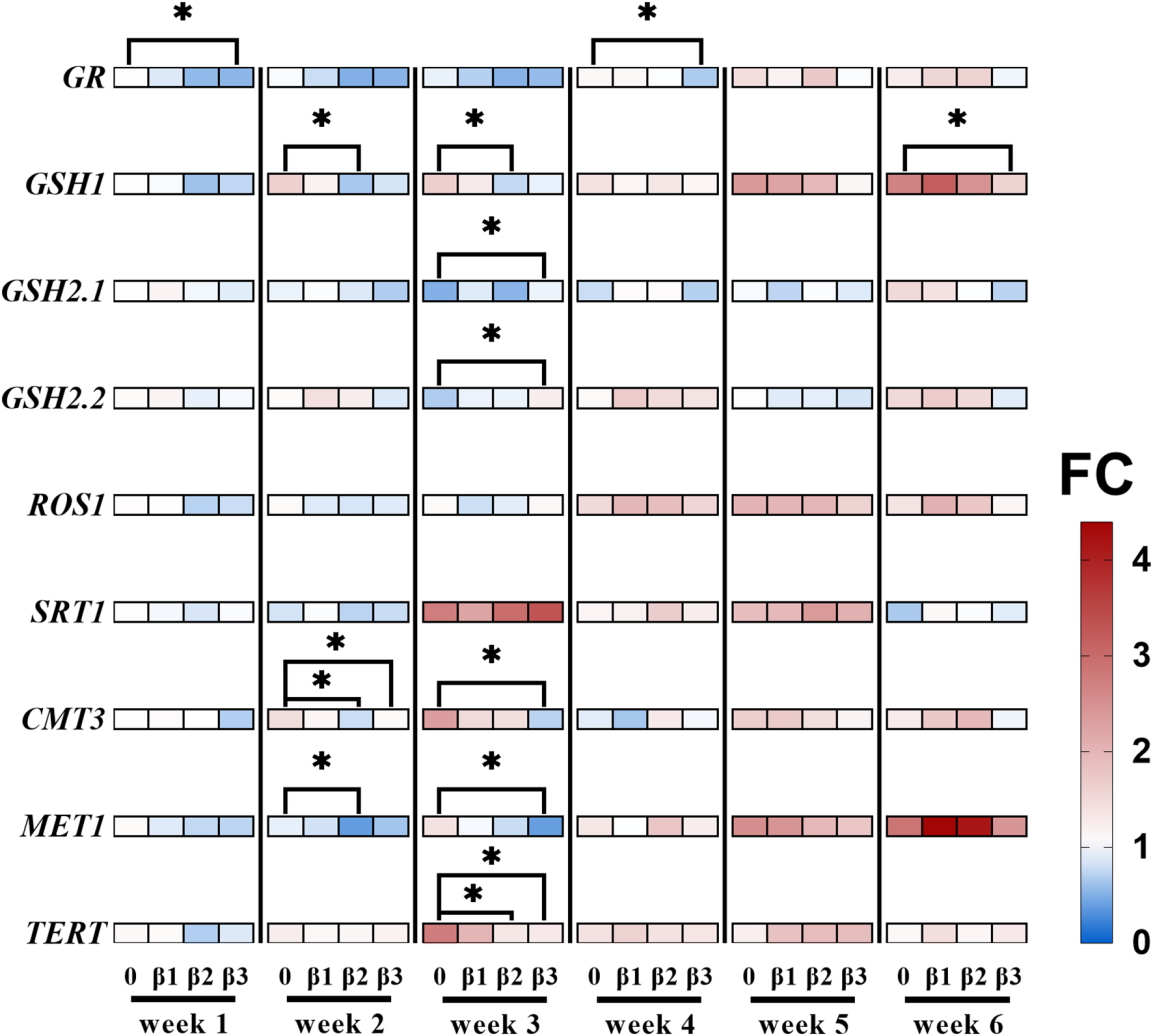
Heatmap representing relative changes of expression for a select set of genes during chronic ^90^Sr exposure. For all data points, the fold change (FC) in gene expression is relative to the control condition of week 1, where red represents increase and blue decrease in gene expression. Data is described following a weekly chronological order, and the full data is represented in **Supplementary Table 1**. Statistical significance is represented by an asterisk (p-value < 0.05 (*), p-value < 0.01 (**), p-value < 0.001 (***), p-value < 0.0001 (****); Wilcoxon test) for the treatment-control pairwise comparison within each week. Plants were cultured in growth medium and exposed to different activity concentrations of IR from a ^90^Sr source. Dose rate values: 0 (control), 0.82 (β1), 7.69 (β2) and 72.67 (β3) µGy•h^-1^. GR, glutathione reductase; GSH1, glutamate-cysteine ligase; GSH2.1, glutathione synthetase 2 variant 1; GSH2.2, glutathione synthetase 2 variant 2; ROS1, repressor of silencing 1; SRT1, sirtuin 1; CMT3, chromomethylase 3; MET1, DNA methyltransferase 1; TERT, telomere reverse transcriptase. µGy, microgray; h, hour.

2DNA methylation is widely known to play a role in the adaptation of plants to chronic IR exposure, modulating the expression of specific genes (Bondarenko et al., 2023; Horemans et al., 2018; Kovalchuk et al., 2003; Laanen et al., 2021; Volkova et al., 2018). Furthermore, glutathione biosynthesis and DNA methylation pathway compete for the common substrate S-adenosylmethionine (SAM) (Ouyang et al., 2020), indicating an interconnection between redox balance and epigenetic regulation. As such, the current work included the evaluation of gene transcript levels that control DNA methylation (repressor of silencing 1 (ROS1), chromomethylase 3 (CMT3), DNA methyltransferase 1 (MET1)), of key enzymes that play crucial roles in plant’s redox metabolism (glutathione reductase (GR), glutamate-cysteine ligase (GSH1), glutathione synthetase 2 variant 1 (GSH2.1), glutathione synthetase 2 variant 2 (GSH2.2)), and chromatin conformation (sirtuin 1 (SRT1), telomere reverse transcriptase (TERT)). The results of the relative gene expression over the weeks are represented in **Figure 6** and **Supplementary Table 1**.

Results indicate that GR expression is decreased in the β3 group in week 1 and week 4, GSH1 expression is lowered in the β2 group in week 2 and 3, and in the β3 group of week 6, suggesting a potential role for glutathione homeostasis in early onset ^90^Sr-induced stress responses. Additionally, week 3 results show an increased expression of GSH2.1 and GSH2.2, suggesting a possible shift in the stress scavenging system involved. For the β2 and β3 conditions, MET1 and CMT3 levels decreased in weeks 2 and 3, respectively, with TERT also showing decreased levels in week 3. These changes suggested a dose rate and time dependent variation in DNA methylation maintenance and telomere regulation during early to intermediate stages of ^90^Sr exposure.

## 4 Discussion

The objective of the present study was to gain insight into the mechanistic process involved in the plant responses to chronic, multi-generational ^90^Sr IR exposure. To this end, the fast-growing aquatic plant *L. minor* was exposed to low-dose ^90^Sr contaminated media. The lowest dose rate (β1, 0.82 ± 0.07 µGy•h^-1^) was chosen based on the ^90^Sr activity concentration found in the lakes of the CEZ (Shevchenko et al., 2001; Teien et al., 2021), habitat of *Lemna* spp, with the final aim of increasing ecological realism. The second dose rate (β2, 7.69 ± 0.98 µGy•h^-1^) retained particular relevance as it could be approximated to the screening value (“SV”, 10 µGy•h^-1^), a generic predicted no-effect dose rate resulting from a species sensitivity distribution analysis of effects data (Andersson et al., 2009). It represented a value below which 95% of the species should be protected from IR (Andersson et al., 2009). The highest dose rate (β3) of 72.67 ± 6.90 µGy•h^-1^, which is still considered to be a low dose rate for *L. minor* (Xie et al., 2019), represented a 10-fold increase of the SV and was chosen based on previous research which demonstrated that it could induce molecular changes in *L. minor* (Van Hoeck et al., 2017).

### 4.1 Non-linear and graded response: dose-rate dependence through the lens of the early-response phase

The available acute (Alkimin et al., 2019; Flores-Rojas et al., 2015; Nunes et al., 2014; Park et al., 2017) and chronic (Radić et al., 2010; Van Hoeck et al., 2017; Xie et al., 2022) IR-related studies in *L. minor* usually cover up to 1 week of exposure, which for such a fast reproduction plant represents about 2 to 3 clonal generations (i.e. ~2.5 days/generation) (OECD, 2006).

With respect to the field of plant radioecology, Volkova et al. (2022) define the hormesis mechanism as biological responses characterized by faster development, more robust growth, greater stress tolerance, or the build-up of specific chemicals in response to low-dose radiation. In the early phase of exposure (week 1), ^90^Sr exposed *L*. *minor* plants showed increased frond area parameters, evident at the lowest dose rates β1 and β2 (**Figure 1**).

The data further suggested that even the low and environmentally relevant doses employed in the study were sufficient for the plants to experience stress. It is important to discern between “*eustress*” and “*distress*”, which are respectively described in *L. minor* as growth preserving or stimulating and growth inhibiting types of stress (Van Hoeck et al., 2017). Van Hoeck et al., demonstrated that “*distress*” after a one-week exposure in *L. minor* begins at dose rates of milligrays per hour (Van Hoeck et al., 2017). Our results suggests that *eustress* can be induced already at much lower dose rates (e.g. 0.82 ± 0.07 µGy•h^-1^, **Table 1**). These results underline the necessity to take into consideration low-dose rates when assessing the ecological impact of radioactive contamination.

### 4.2 Long-term and time-dependent responses

The experimental design employed in the present work covered approximately 17 *L. minor* generations. Here, time dependence refers to the variation in treatment responses across weeks relative to their respective within-week controls. The results suggest a temporal pattern of response to low-level chronic ^90^Sr contamination that may be interpreted as comprising three phases over the 6-week period: the above described early phase (week 1), the mid phase (weeks 2-5), and the long-term phase (week 6). In the mid-phase, the main observable endpoint changes was frond number, which increased consistently throughout all the weeks (**Figure 2**), and a decline in pigment content (**Figure 3**). Interestingly, the decrease in pigment content coincided with conditions and time points where frond number increased. On the other hand, the long-term response phase was characterised by a combination of biomarker changes that mirrored week 1 (see **Table 3**). In fact, early and long-term phases shared increased levels of area average specific frond rate (**Figure 1**), decreased starch content (**Figure 4**) and decreased GPOD (**Figure 5**) activity values. However, one difference must be mentioned: while in week 1 only dose rates β1 and β2 were showing increased area ASGR, in week 6 β3 plants showed significant increased values as well. This might be suggesting the presence of a dose rate and time dependent transient hormetic effect, with the plants taking more time to acclimate to higher dose rates.

**Table 3:** Table representing endpoints changes in a 6-week chronic ^90^Sr exposure experiment.

Endpoints		Weeks					
		Week 1	Week 2	Week 3	Week 4	Week 5	Week 6
		Early phase	Mid phase				Long-term phase
Telomere length							
Electron transport rate			↓β3 at 435 PAR				
Glutathione				**∅**	**∅**	**∅**	
Fresh weight					↑β3		↑β3
Dry weight				↑β2			↑β3
Area ASGR		↑β1, β2			↑β3		↑β1, β2, β3
Frond number ASGR			↑β1, β2	↑β2	↑β1, β2, β3	↑β1, β2, β3	
Chlorophyll a			↓β1, β2		↓β1, β2, β3		
Chlorophyll b			↓β2		↓β1, β2, β3		
Chla/Chlb		↑β3			↑β3		
Carotenoids			↓β2		↓β1, β2, β3		
GPOD activity		↓β1, β2, β3		**∅**	**∅**	**∅**	↓β1, β2, β3
Total protein						↑β3	
Total sugar			↑β2	↑β1			
Starch		↓β3		↓β1	↓β1	↓β1	↓β1
Gene expression	*GR*	↓β3			↓β3		
	*GSH1*		↓β2	↓β2			↓β3
	*GSH2.1*			↑β3			
	*GSH2.2*			↑β3			
	*ROS1*						
	*SRT1*						
	*CMT3*		↓β2, β3	↓β3			
	*MET1*		↓β2	↓β3			
	*TERT*			↓β2, β3			

Plants were cultured in growth medium and exposed to different activity concentrations of IR from a ^90^Sr source. Dose rate values: 0, (β1) 0.82, (β2) 7.69 and (β3) 72.67 µGy•h^-1^. Upward and downward pointing arrows respectively refer to increased and decreased values compared to the control samples. ∅, non-tested weeks; GR, glutathione reductase; GSH1, glutamate-cysteine ligase; GSH2.1, glutathione synthetase 2 variant 1; GSH2.2, glutathione synthetase 2 variant 2; ROS1, repressor of silencing 1; SRT1, sirtuin 1; CMT3, chromomethylase 3; MET1, DNA methyltransferase 1; TERT, telomere reverse transcriptase.

Through rapid expansion on the water surface, *L. minor* plants can outcompete other species for access to light and nutrients, effectively monopolizing the available space and resources (Paolacci et al., 2018). Therefore, producing additional fronds quickly under *eustress* conditions might represent a competitive advantage over other aquatic plants. The observed increase in frond number ASGR during the mid-phase lends support to this hypothesis. However, the relative importance of frond number and area in *L. minor*’s competitive strategies may vary depending on the specific environmental context (Vasseur et al., 1995).

It was evident that there were re-adjustments in total macromolecular content and in the antioxidant photosynthetic pigment repertoire, as increases in total protein content (**Figure 4A**) were detected in week 5, increases in soluble sugar content (**Figure 4B**) were measured in weeks 2 and 3, decreases in starch content (**Figure 4C**) were seen in weeks 1 and 3-6, and decreases in the photoactive pigments chlorophyll a, b and carotenoids were respectively observed in weeks 2 and 4, in weeks 2-4, and weeks 2 and 4 (**Figure 3**). Similarly to what happens for frond number ASGR, total macromolecular content and pigment results were chiefly dose rate- and time-dependent. In particular, pigment levels appeared to be decreased in the lowest dose rates (β1 and/or β2) when the first detectable difference emerged in week 2, while all the dose rates showed decreased levels in week 4. Another indication of this time- and dose rate-dependence is that, from week 3 to week 6, only the lowest dose rate (β1) consistently showed decreased levels of starch content. This finding is in line with the current literature reporting that the time necessary for the organism to acclimate is relative to the dose rate into play (Shadley and Wiencke, 1989; Tapio and Jacob, 2007).

### 4.3 Indications of dose rate and time dependent transcriptional regulation of the DNA methylation machinery and glutathione metabolism genes

Biological systems use a range of feed-forward regulatory processes to preserve epigenetic information for prolonged periods of time (Castel and Martienssen, 2013; Houri-Zeevi and Rechavi, 2017; Martienssen and Moazed, 2015). Among them, we can find the IR-responsive mechanisms (Laanen et al., 2023) of DNA methylation regulation (Agorio et al., 2017). In this study, the expression of several genes involved in DNA methylation regulation was tested, namely DNA Methyltransferase 1 (*MET1*) and Chromomethylase 3 (*CMT3*), which are essential to the maintenance of DNA methylation (Chan et al., 2005), and Repressor of Silencing 1 (*ROS1*), a demethylation agent that prevents excessive methylation and ensures that epigenetic changes are precisely calibrated to the needs of the plant (Halter et al., 2021, p. 1). In addition to DNA methylation, histone modifications epigenetically regulate chromatin structure and function (Miller and Grant, 2013) and are reported to be involved in plants’ IR stress responses (Kim et al., 2019; Raut and Sainis, 2012). Thus, the expression analysis of the histone deacetylase *SIRT1* gene, an epigenetic regulator involved in *A. thaliana* abiotic stress responses (Liu et al., 2017), was included in this study as well. The beginning of the mid phase was marked by an overall change in gene expression of the DNA methylation machinery: while methyltransferases genes showed decreased expression for dose rates β2 and β3, no changes in gene expression were observed for the demethylase *ROS1* across all dose rates (**Figure 6**; **Supplementary Table 1**). Similarly to what Van Antro et al. proposed in their work on the involvement of DNA methylation in *L. minor*’s temperature stress responses, these results suggested a potential dose- and time-dependent involvement of the epigenetic machinery in IR stress response in *L. minor*. For further elucidation of the role of epigenetics in this stress response over time, the current analysis should be complemented by western blotting and/or bisulfite sequencing to provide a more comprehensive understanding.

The DNA methylation machinery relies on S-adenosyl methionine (SAM) to exert its functions (Ouyang et al., 2020), and SAM plays a which also constitutes an important precursor for the synthesis of glutathione. Here, the relative expression of *Glutamate-cysteine ligase* (*GSH1*), *Glutathione synthetase 2 variant 1* (*GSH2*.1), *Glutathione synthetase 2 variant 2* (*GSH2*.2), *Glutathione reductase* (*GR*) was followed over the course of the 6 weeks exposure. Interestingly, despite non-significant decreasing trends of glutathione content (**Supplementary Figure 7**), the expression patterns of glutathione metabolism genes showed significant variations. Specifically, *GR* expression decreased in β3 conditions in weeks 1 and 4, *GSH1* decreased in β2 conditions in weeks 2 and 3 and in β3 conditions in week 6, whereas *GSH2*.1 and *GSH2*.2 increased under β3 conditions in week 3 (**Figure 6**). Thus, the mid phase showed the lion’s share of responses at the gene expression levels for glutathione metabolism genes for β2- and β3-exposed plants. Just as it happened with epigenetic expression data, they went back to normal levels upon progressing towards the long-term responses phase, highlighting again the long-term transient nature of these responses.

### 4.4 Transient regulation of *TERT*: the enigma of telomere dynamics in the context of chronic IR responses

It has been reported that telomere regulation is involved in plant stress responses and aging (McKnight et al., 2002; Sun et al., 2023; Watson and Riha, 2010). Works from Wyatt and Shippen (Ohio.edu, 2022; Russel, 2022) report that plants grown in space, where the background IR levels are higher [e.g. daily dose on the International Space Station is 0.5 mSv, which is about 100 higher than the dose on earth (Cucinotta et al., 2008; Takahashi et al., 2018 De Micco et al., 2023)], have a steady telomere length but significant changes in telomerase enzyme activity.

In our work, we carried out the measurement of telomere length, and analysed *TERT* (*Telomerase Reverse Transcriptase*) transcript accumulation, essential for telomere length maintenance (Vaquero-Sedas and Vega-Palas, 2014). The qRT-PCR results of pointed out a time- and dose-dependence in the mid phase, as only β2 and β3 conditions appeared to be downregulated and only in week 3 (**Figure 6**). In contrast to this result, the telomere length assessment showed stable levels throughout the experiment (**Supplementary Figure 4**). As the telomerase system is intimately linked to the epigenetic machinery, chromatin 3D structure and DNA methylation in particular (Dogan and Forsyth, 2021; Galati et al., 2013; Jezek and Green, 2019; Moores et al., 2011), further research investigating this relationship could provide valuable insights into the role of the telomerase system in the plant’s response to low level ^90^Sr radiation.

## 5 Conclusion and perspectives

This study examined the biological responses of *Lemna minor* to chronic low-dose ^90^Sr exposure over a six-week period, covering approximately 17 clonal generations. The data revealed time- and dose-dependent responses, which could be interpreted as early-, mid-, and long-term. These included transient hormetic effects on frond area and number, significant shifts in macromolecular (sugars and proteins) and photoactive pigment levels. At the molecular level, changes were observed in the expression of genes involved in DNA methylation, glutathione metabolism, and telomere maintenance, while telomere length itself remained stable throughout.

Taken together, these findings provide new data on how *L. minor* responds at multiple biological levels under environmentally relevant chronic exposure to ^90^Sr. The study contributes to the understanding of plant acclimation strategies to ionizing radiation, with potential implications for phytoremediation and the development of more refined chronic exposure endpoints in environmental risk assessment frameworks.

## Supplementary methods

### Uptake of ^90^Sr

At day 7 of each experimental week, after subculturing, all plants growing in 3 pots were collected for measuring ^90^Sr uptake. To wash off any excess ^90^Sr that could have adhered to the plants, they were transferred to a small sieve and underwent 3 washing steps: 10 minutes in 10 mL 1mM Pb(NO_3_)_2_ and 2 steps of 10 minutes in 15 mL demineralized water. Plants were then dried and transferred to a weighing vessel for fresh weight (FW) measurement. Plant material was then placed in an oven at 65°C until dry. Plant samples were then weighed (dry weight, DW) and transferred to 20 mL glass vials, where they were ashed for 24h. The internal temperature of the oven was reached in small steps (from 0 to 550°C in 12h) to avoid losing material from the sample. After the samples were completely ashed, the dissolution procedure started by adding 1 mL of 65% nitric acid (HNO_3_). Vials were placed on a sand bath until dry, then moved to the oven and left at 550°C overnight. Subsequently, plants were mineralized by adding a solution of 65% HNO_3_ and 30% hydrogen peroxide (H_2_O_2_) in three sequential septs: 0.65 mL, 0.65 mL and 0.70 mL. The samples were then dried, after which 2 mL of 65% HNO_3_ was added. This stepwise addition prevented liquid loss due to bubble formation during the process. The drying step was repeated as needed to safely add the full volume required for complete mineralization. The samples were then dried again to ensure thorough preparation for the subsequent analysis. Finally, samples were dissolved in 8 mL of 0.05 M HNO_3_, transferred to polyethylene (PE) vials, weighed, and 12 mL of scintillation cocktail (Optiphase Hisafe 3) were added. β measurement was carried out by the Low-level Radioactivity Measurements (LRM) group at SCK CEN (Belgium) employing the following protocol: reference and blank samples were prepared in the same way as samples. The reference solution, a dilution from a certified reference solution from CERCA-LEA, contained a known amount of solution of ^90^Sr/^90^Y (80.68 ± 2.42 Bq ^90^Sr). The samples were kept for one hour in the counter before starting the measurements. The blank and the reference were measured together (1h per vial) with the samples. A Hidex 300SLL liquid scintillation counter was used to perform the measurement.

### Dosimetry

Dose rate values [µGy•h^-1^] were computed by the ERICA tool (Brown et al., 2008) and calculations were based on the “*Lemna* leaf model”. Fronds were modelled as elliptical cylinders adopting the standard parameters of *L. minor* geometry described in Van Dyck et al (Van Dyck et al., 2021), which featured dimensions of 4.1 mm in length, 2.7 mm in width, and 0.4 mm in height, with an average frond mass of 1.66 mg. The dosimetry focused solely on the plants’ frond, with the root component being disregarded due to its relatively low dose contribution. For external dose calculations, the plant was considered to be “on water”, indicating its position as floating on the surface at the centre of the pot. Total dose rates were obtained by adding the internal and external dose rate values given by the ERICA tool. For each week and contamination condition, concentration factors (CF) were calculated and implemented according to the following equations:

1. DR_int_ = A_w_ • CF • DC_int_ [µGy•h^-1^]
2. DR_ext_ = A_w_ • DC_ext_ [µGy•h^-1^]
3. DR_total_ = DR_int_ + DR_ext_ = A_w_ • CF • DC_int_ + A_w_ • DC_ext_ = A_w_ • (CF • DC_int_ + DC_ext_) ≈ A_w_ • CF • DC_int_

where A_w_ is activity in the growing medium [Bq•L^-1^], CF is concentration factor [L•kg^-1^], DC_int_ is internal dose conversion coefficient [µGy•h^-1^ per Bq•kg^-1^], DC_ext_ is external dose conversion coefficient [µGy•h^-1^], DR_int_ is internal dose rate [µGy•h^-1^], DR_ext_ is external dose rate [µGy•h^-1^], DR_total_ is total dose rate [µGy•h^-1^].

### DNA extraction

DNA was extracted by first grinding 100 mg of frozen sample material, adding 500 µL of CTAB lysis solution (100mM Tris-HCl pH 7.5, 2% CTAB, 1.4M NaCl, 20mM EDTA pH 7.5, 1% NaHSO_3_), and then incubating at 65°C for 30 minutes. Next, 500 µL of a 24:1 mixture of chloroform:IAA was added. After a gentle inversion, centrifugation at 12,000 g for 15 minutes followed. After carefully transferring the upper aqueous layer to a fresh tube, isopropanol precipitation step was performed by adding 350 µL of isopropanol, following incubation at -20°C for 15 minutes. The DNA pellet was spun down again (12,000 x g for 30 min) and the supernatant was removed before being washed with 70% ethanol and air-dried. The freshly extracted DNA was then obtained by resuspending the DNA pellet in 50 µL of water, making it available for further molecular analysis. DNA concentration was measured using the NanoDrop ND1000 and DNA integrity was assessed through gel electrophoresis (1% Agar, Tris-Acetate-EDTA (TAE) buffer).

### Gene expression analysis by quantitative real-time PCR

Liquid nitrogen-frozen plant samples (50 mg) were shred using 2.0 mm Zirconia (zirconium (IV)oxide) beads (BioSpec Products) in a Mixer Mill MM 400 (Retsch) for 3 min at 30 Hz. The RNA was extracted using the RNeasy^®^ Plant Mini Kit (Qiagen, reference 74904), as recommended by the manufacturer. The NanoDrop ND1000 was then used to perform spectrophotometric measurements of RNA quality and quantity. Contaminant DNA was removed with the Thermo Fisher Scientific’s InvitrogenTM TURBO DNA freeTM kit (INVITROGEN, reference AM1907) and TaKaRa Bio’s PrimeScript 1st strand cDNA synthesis kit (TaKaRa Bio, reference 6110A), was used for cDNA synthesis, following the instructions of the manufacturers.

Gene expression analysis was performed by quantitative real-time PCR (qRT-PCR) on a 96-wells QuantStudio3 (Applied Biosystems) using Quantinova^®^ SYBR^®^ Green PCR kit (Cat. No. 208056). The qRT-PCR workflow was developed in accordance with the Eleven Golden Rules of quantitative RT-PCR (Udvardi et al., 2008), ensuring data quality and reliability. The analysis was run in 40 cycles using the following program: 5 seconds at 95°C, 12 seconds at 60°C. Comparison of the expression profile of five potential housekeeping genes, namely *CYP, MAP2K1, BSL2, SBT3.3* and *TUBB5* indicated that the latter was the most stable one across time-points and conditions, and it was used for normalizing target gene levels by the delta-Ct method employing the RefFinder tool (Xie et al., 2012) (see **Supplementary Figure 8**). The selected genes were identified by NCBI BLAST searches (Altschul et al., 1990) of *A. thaliana* genes against *L. minor* (serial number 1007 and ID number 5500) genome (Van Hoeck et al., 2015a). Additionally, we performed *in silico* validation of each primer pair to confirm sequence specificity both NCBI BLAST against *L. minor*’s transcriptome and the uMelt tool (Dwight et al., 2011). Primer sequences are reported in the **Supplementary Table 2**. Relative gene expression levels were calculated using the comparative Ct (ΔΔCt) method (Livak and Schmittgen, 2001). First, the Ct values of target genes were normalized to *TUBB5* to obtain ΔCt values. Then, the ΔΔCt was calculated by comparing the ΔCt of each treatment sample to that of the week 1 control. Fold changes in gene expression were subsequently derived using the formula 2^–ΔΔCt^.

### Photosynthetic activity

*L. minor* plants (5–6 plants per condition, 4 replicates) were harvested, transferred to a 6 well plate with 7.5 mL H_2_O demineralized per each well, packed in aluminium foil and let in the dark for at least one hour to ensure dark adaptation. Photosynthetic activity was then analysed using the DUAL-PAM-100 (Walz) machine and its associated software (Dual PAM version 1.19). Measurements were carried out in the dark and included both induction curve (IC) and rapid light curve (RLC) analyses. Immediately prior to the analysis, the measuring cuvette was filled with demineralised water and just enough plants to cover the surface. The full measurement between two different samples was conducted every 15 minutes, resulting in 4 measurements per hour (Schreiber, 2004).

### Pigment measurement

Dimethylformamide (DMF), at a ratio of 0.5 mL per 20 mg of fresh plant material, was used to extract the pigments from each sample, which were then incubated overnight at 4°C in the dark. The absorbance was measured in triplicate at wavelengths of 664, 647, and 480 nm, using 1:1, 1:2, and 1:4 dilutions. Pigment concentrations (carotenoids, chlorophyll a and chlorophyll b) were obtained following the calculations of Wellburn (1994).

### Glutathione measurement

The spectrophotometric measurement of oxidized (GSSG) and reduced (GSH) forms of glutathione was conducted as previously described (Queval and Noctor, 2007; Griffith, 1980; Tietze, 1969). Frozen plant samples (50–80 mg FW) were homogenized while kept frozen with 3 zirconia beads in a Retsch Mixer Mill MM400 at 30 Hz for 3.5 minutes. The measurements were carried out following the modifications previously reported (Horemans et al., 2014).

### Guaiacol peroxidase enzyme capacity

Plant tissue samples (50–80 mg FW) were stored at -80°C prior to analysis. Samples were homogenized with 20 chrome steel beads (Qiagen) and 2 mg of polyvinylpyrrolidone (PVP) in a Retsch Mixer Mill MM400 at 30 Hz for 3 minutes. Subsequently, 750 μL of extraction buffer (0.1 M TRIS; 1 mM Na2-EDTA; 1 mM DTT, pH 7.8) were added and the samples were centrifuged at 13000 rpm for 10 minutes at 4°C. To determine the Guaiacol peroxidase (GPOD) capacity, a solution comprising 110 μL of 0.1 M phosphate buffer, 10 μL of sample extract, and 80 μL of guajacol mastermix, composed of 90 mM guaiacol and 163 mM H_2_O_2_ mixed in a 1:1 ratio. This mixture was added to each well of a plastic 96-well plate. The kinetic monitoring of the appearance of tetraguajacol was conducted at 436 nm using the Biotek PowerWave XS Microplate Reader.

### Telomere length measurement

Telomere length was estimated by qRT-PCR following the protocol from Vaquero-Sedas et al (Vaquero-Sedas and Vega-Palas, 2014). The amplification was performed on a Rotor-Gene Q cycler (QIAGEN), using 5 ng of genomic DNA per vial, 1 µL of each primer (forward and reverse) at a concentration of 10µM, and 10µL of QuantiFast mix with SYBR (QIAGEN, catalog No. 204156) in a final volume of 20 µL. The program was set for holding for 15 minutes at 95°C. Then, 15 seconds at 94°C followed by 15 seconds at 49°C, for two cycles. This was followed by 32 amplification cycles consisting of 94°C for 15 seconds, 62°C for 10 seconds, 74°C for 15 seconds (with signal acquisition for telomeric Ct values), 84°C for 10 seconds, and 85°C for 15 seconds (with signal acquisition for the housekeeping gene). A final extension step was performed at 72°C for 60 seconds. The melt curve analysis followed, with pre-melt conditioning at 72°C for 90 seconds, then a ramp from 72°C to 95°C in 1°C increments with 5 seconds of hold at each step. Each run includes a standard curve of the single-copy housekeeping gene *TUBB5* (Zhang et al., 2017) and for the telomeres that were created by serially diluting the total pool of DNA samples from 2 to 0.6 ng. Primers are listed in **Supplementary Table 2**. Samples from different stages of exposure were distributed in the same run, and reactions were carried out in triplicate, ensuring that the difference in Ct values between duplicates was kept to less than 0.3. The standard curve’s efficiencies for telomeres and tubulin were both within the permissible standard range. One product per pair of primers was amplified, according to analysis of the melting curve. For each specific condition, the ratio (T/S) of the number of telomere tandem copy repeats to the number of housekeeping genes, was used to calculate telomere shortening. Following the approach described by Vaquero-Sedas et al., relative telomere length was determined using the comparative Ct (ΔΔCt) method (Vaquero-Sedas and Vega-Palas, 2014). For each sample, Ct values were obtained for the telomeric sequence and the single-copy reference gene TUBB5 in separate reactions. The ΔCt was calculated as Ct_TEL – Ct_TUBB5. A calibrator sample (control condition) was included on every plate to normalize across runs. The ΔΔCt was then obtained by subtracting the calibrator’s ΔCt from the sample’s ΔCt. Finally, the relative telomere length was expressed as 2^–ΔΔCt^. All reactions were performed in technical duplicates, and primer efficiencies were confirmed to be within the acceptable range.

### Protein content measurement

First, the frozen plant samples in Eppendorf tubes were ground to a fine powder with 15 chrome steel beads (Qiagen) in a Retsch Mixer Mill MM400 at 30 Hz for 3 minutes. 100 mL of extraction buffer were prepared as follows: Tris-HCl (1M pH 8.8) 12 mL, EDTA (0.25 M) 1.6 mL, SDS (10%) 40 mL, glycerol (50%) 20 mL, and distilled H_2_O up to 100 mL. 2 mL of buffer were added to the samples, they were mixed well then centrifuged at 12000 rpm for 20 min at 4°C. The supernatant liquids containing total proteins fraction (protein extract) were transferred to clean Eppendorf tubes and stored at -20°C. This total protein content measurement was then carried out following the Bio Rad DC™ protein assay protocol: Reagent A’ was prepared by adding 20 µL of reagent S (C_12_H_25_NaO_4_S 7%) to each mL of reagent A (NaOH 3%). A standard curve was prepared using a protein standard diluted to concentrations ranging from 0.2 mg/mL to 1.5 mg/mL. 5 µL of standards and samples were pipetted into a clean, dry microplate, followed by the addition of 25 µL of reagent A’ and 200 µL of reagent B (a diluted FOLIN reagent containing less than 1% each of the following ingredients: lithium sulfate, tungstic acid, sodium salt, molybdic acid, hydrochloric acid and phosphoric acid). After a 15-minute incubation at room temperature, absorbance was measured at 750 nm using the Biotek PowerWave XS Microplate Reader. The protein concentration of *L. minor* samples was determined by plotting absorbance against the concentration of known standards and interpolating the absorbance values of unknown concentration samples.

### Sugar and starch content measurement

Plant samples were dried in an oven at 60°C for 3 days. Around 15 mg of dried biomass were weighed into a 2 mL Eppendorf tube and mixed with 3 chrome steel beads. Samples were then shredded using a Mixer Mill MM400 at 30 Hz for 4 minutes. Interfering pigments were extracted with 100% acetone: four extractions of 1 mL each were performed, followed by vortexing and centrifugation for 1.5 minutes at maximum speed. The beads and the supernatant were removed. Sugars were extracted with 80% ethanol: two extractions of 1 mL each were performed, and the entire pellet was transferred to a 15 mL tube for starch extraction, where 3 mL of 80% ethanol were then added. The samples were then centrifuged for 5 minutes at maximum speed (4800 rpm) using a Rotina 420R centrifuge. The supernatant containing soluble sugars was transferred to a new 15 mL tube labelled for sugar extraction. Starch residues were hydrolysed through an addition of 5 mL of 1.1% HCl. The samples were heated in a water bath at 100°C for 30 minutes. 5 mL of demineralized H_2_O were added to each sample, resulting in a total volume of 10 mL. Before the spectrophotometric measurements, the following solutions were prepared: anthrone reagent was prepared adding 0.1 g anthrone (C_14_H_10_O) in 50 mL of 72% H_2_SO_4_, starch standards (0 to 10 mg starch/10 mL 1,1% HCl), and glucose standard (0 to 10 mg glucose/10 mL 80% EtOH). The concentration of unknown samples was analysed as follows: 100 mL of test solution (samples or standard solutions) were pipetted into a 2 mL Eppendorf tube and cooled to 0°C on ice. Then, 500 µL of ice-cold anthrone reagent were added, and all samples were vortexed. The samples were then heated for exactly 11 minutes in a water bath at 100°C, cooled rapidly to 0°C on ice, and vortexed again. Absorbance was read at 630 nm using the Biotek PowerWave XS Microplate Reader within an hour. The concentration of starch and glucose of *L. minor* samples were determined by plotting absorbance against the concentration of known standards and interpolating the absorbance values of unknown concentration samples.

## Data Availability

The original contributions presented in the study are included in the article/**Supplementary Material**, further inquiries can be directed to the corresponding authors.

## Supplementary material

Supplementary Figure 1 Volume minimization pilot experiment (area ASGR in function of number of plants and volume of Hoagland solution). Overview of frond area ASGR changes after 7 days of growth for 4 different numbers of plants (3, 5, 7, or 9 plants) grown in a single pot and 3 different volume conditions (25, 50, and 100 mL). Data are presented as mean ± SD (n=3). Plants were cultured in 1/10 strength Hoagland solution without contaminants. Statistical significance between 50 mL and the other volume conditions was tested with two-way ANOVA. No statistically significant differences were observed (ns).

Supplementary Figure 2 Absolute values frond area. Overviews of frond area changes at day 0, day 2, day 4 and day 7. Data are presented as mean ± SD (n=15). Plants were cultured in growth medium and exposed to different activity concentrations of IR from a ^90^Sr source. Dose rate values: 0, 0.82, 7.69 and 72.67 µGy•h^-1^. Within week statistical significance is represented by * (significance levels: p-value < 0.05 (*), p-value < 0.01 (**), p-value < 0.001 (***), p-value < 0.0001 (****); two-way ANOVA). µGy = microgray, h= hour.

Supplementary Figure 3 Absolute values frond number. Overviews of frond number changes at day 0, day 2, day 4 and day 7. Data are presented as mean ± SD (n=15). Plants were cultured in growth medium and exposed to different activity concentrations of IR from a ^90^Sr source. Dose rate values: 0, 0.82, 7.69 and 72.67 µGy•h^-1^. Within week statistical significance is represented by * (significance levels: p-value < 0.05 (*), p-value < 0.01 (**), p-value < 0.001 (***), p-value < 0.0001 (****); two-way ANOVA). µGy = microgray, h= hour.

Supplementary Figure 4 Telomere length. Relative telomere length across weeks. Data are presented as mean ± SD (n=3). Plants were cultured in growth medium and exposed to different activity concentrations of IR from a ^90^Sr source: 0, 0.82, 7.69 and 72.67 µGy •h^-1^. Within week statistical significance is represented by * (significance levels: p-value < 0.05 (*), p-value < 0.01 (**), p-value < 0.001 (***), p-value < 0.0001 (****); two-way ANOVA).

Supplementary Figure 5 ETR dynamics. **(A-F)** Electron transport rate of weeks 1–6 plotted against PAR irradiance values. Data are presented as mean ± SD (n=3). Plants were cultured in growth medium and exposed to different activity concentrations of IR from a ^90^Sr source. Dose rate values: 0 (control), 0.82 (β1), 7.69 (β2) and 72.67 (β3) µGy•h^-1^. Within week statistical significance is represented by the appropriate dose rate symbol (e.g. β3) coupled with an asterisk (significance levels: p-value < 0.05 (*), p-value < 0.01 (**), p-value < 0.001 (***), p-value < 0.0001 (****); two-way ANOVA).

Supplementary Figure 6 Chlorophyll a to chlorophyll b ratio. Chlorophyll a to chlorophyll b ratio fluctuations. Data are presented as mean ± SD (n=3). Plants were cultured in growth medium and exposed to different activity concentrations of IR from a ^90^Sr source: 0, 0.82, 7.69 and 72.67 µGy •h^-1^. Within week statistical significance is represented by * (p-value < 0.05; two-way ANOVA). µGy = microgray, h= hour.

Supplementary Figure 7 Glutathione measurements. Overviews glutathione levels in samples from week 1, week 2 and week 6. Data are presented as mean ± SD (n=4). Plants were cultured in growth medium and exposed to different activity concentrations of IR from a ^90^Sr source. Dose rate values: 0, 0.82, 7.69 and 72.67 µGy•h^-1^. **(A)** Total glutathione content; **(B)** Reduced glutathione content; **(C)** Oxidized glutathione content; **(D)** Percentage of oxidized glutathione. Statistical significance is represented by * (significance levels: (p-value < 0.05 (*), p-value < 0.01 (**), p-value < 0.001 (***), p-value < 0.0001 (****); two-way ANOVA). µGy = microgray, h= hour.

Supplementary Figure 8 Comprehensive stability of housekeeping genes. Graph showing the comprehensive stability ranking of reference genes (*TUBB5, CYP, MAP2K1, BSL2*, and *SBT3.3*) based on the geometric mean of their ranking values. Stability assessment was performed using RefFinder, a web-based tool that integrates geNorm, NormFinder, BestKeeper, and the comparative ΔCt method to assign weighted rankings to each gene. Lower geomean values indicate higher expression stability. *TUBB5 = Tubulin beta-5 chain; CYP71A25 = Cytochrome P45071A25; MAP2K1 = Mitogen-activated protein kinase kinase 1; BSL2= Serine/threonine-protein phosphatase; SBT3.3 = Subtilisin-like protease.*

Supplementary Figure 9 Agarose gel electrophoresis of PCR products for genes of interest. PCR products of genes employed in the RT-qPCR analysis were separated on a 2% agarose gel stained with ethidium bromide and visualized under UV light. Lane A: GeneRuler Low Range DNA Ladder (Cat. No. SM1193, ThermoScientific). Lanes 1–10 correspond to the following genes: 1. *DNA methyltransferase 1 (MET1)*. 2. *Chromomethylase 3 (CMT3)*. 3. *Repressor of silencing 1 (ROS1)*. 4. *Sirtuin 1 (SRT1)*. 5. *Glutamate-cysteine ligase (GSH1)*. 6. *Glutathione synthetase 2 variant 1 (GSH2.1)*. 7. *Glutathione synthetase 2 variant 2 (GSH2.2)*. 8. *Glutathione reductase (GR)*. 9. *Telomere reverse transcriptase (TERT)*. 10. *Tubulin beta-5 chain (TUBB5)*. All genes show single bands at the expected sizes, confirming the specificity of the amplified PCR fragments.

Supplementary Table 1 Table representing qRT-PCR data: changes in gene expression (average fold change) during chronic Sr-90 exposure. Data are presented as mean ± SD (n=4). Plants were cultured in growth medium and exposed to different activity concentrations of IR from a Sr-90 source. Dose rate values: 0, 0.82, 7.69 and 72.67 µGy•h^-1^. *GR = glutathione reductase*; *GSH1 = glutamate-cysteine ligase; GSH2.1 = glutathione synthetase 2 variant 1*; *GSH2.2 = glutathione synthetase 2 variant 2; ROS1 = repressor of silencing 1; SRT1 = sirtuin 1; CMT3 = chromomethylase 3; MET1 = DNA methyltransferase 1; TERT = telomere reverse transcriptase.* Timepoints with a significant effect of Sr-90 exposure on gene expression are represented by * (p-value < 0.05; Wilcoxon Rank-Sum Test). µGy = microgray, h= hour.

Supplementary Table 2 List of primers employed in the present work. Names, functions, and primer sequences are presented.
