# Supplementary figures and images for: Chronic low dose 90Sr contamination in Lemna minor: from transcriptional dynamics of epigenetic regulators to population level effects
Source: Front Plant Sci. 2025 Jun 26;16:1605017. doi: 10.3389/fpls.2025.1605017 (PMC12243032; doi:10.3389/fpls.2025.1605017)

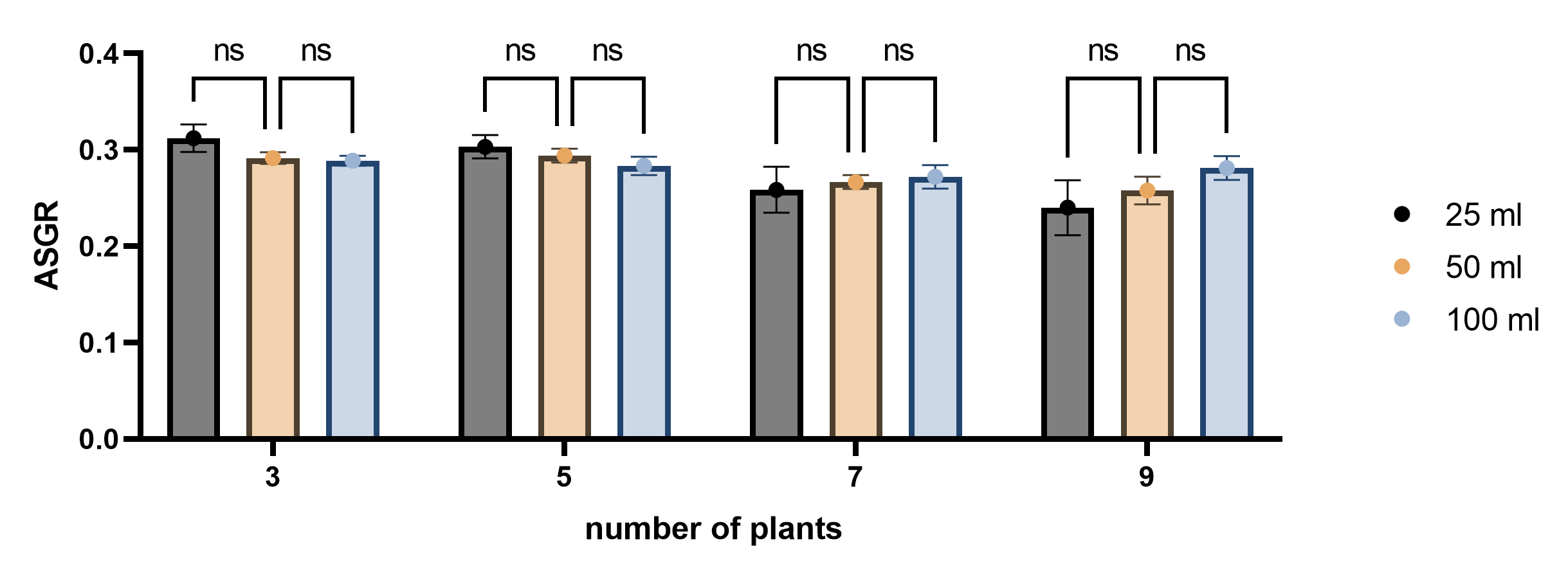

Supplement: Supplementary Figure 1 — Volume minimization pilot experiment (area ASGR in function of number of plants and volume of Hoagland solution). Overview of frond area ASGR changes after 7 days of growth for 4 different numbers of plants (3, 5, 7, or 9 plants) grown in a single pot and 3 different volume conditions (25, 50, and 100 mL). Data are presented as mean ± SD (n=3). Plants were cultured in 1/10 strength Hoagland solution without contaminants. Statistical significance between 50 mL and the other volume conditions was tested with two-way ANOVA. No statistically significant differences were observed (ns). [file Image1.tif]

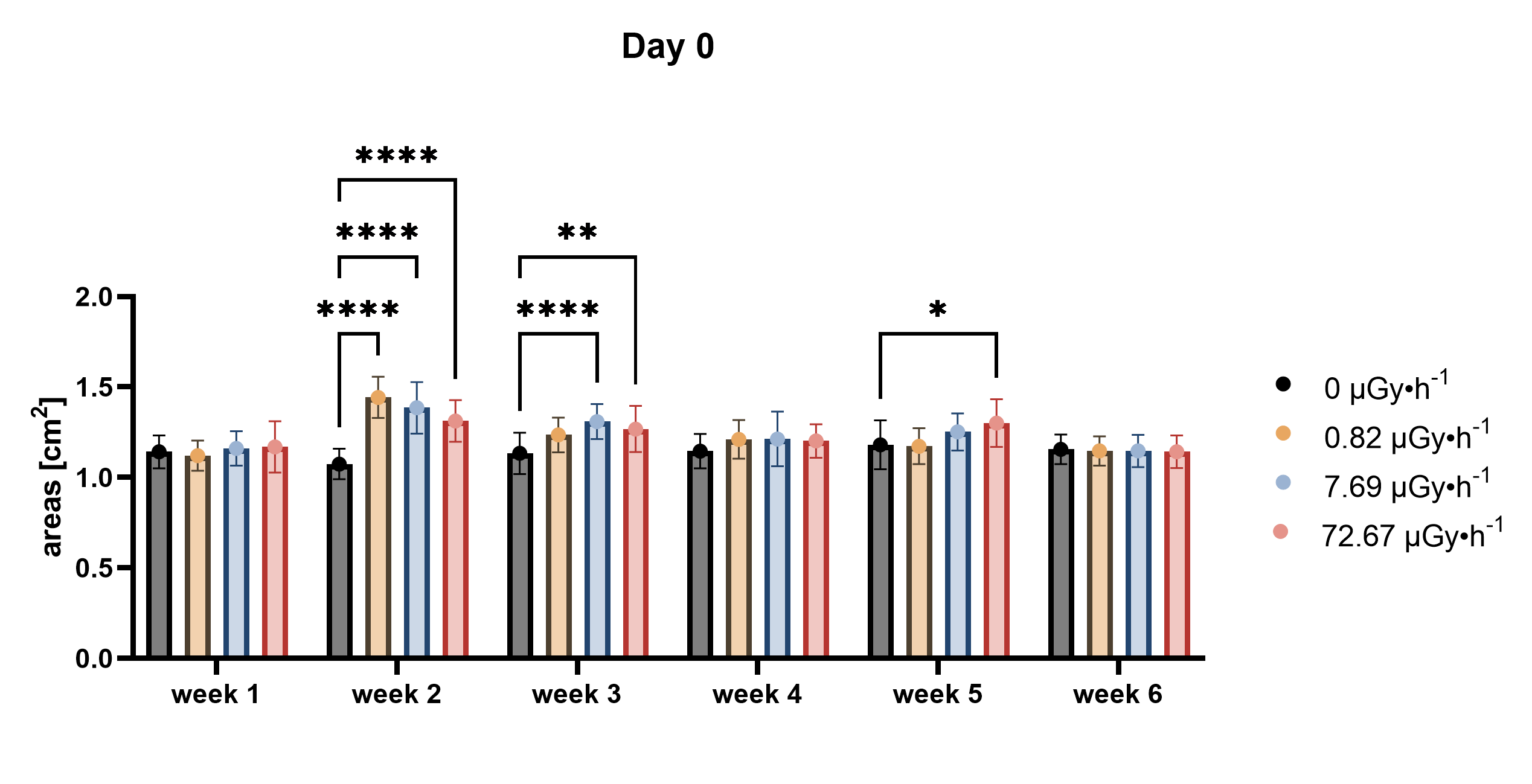

Supplement: Supplementary Figure 2 — Absolute values frond area. Overviews of frond area changes at day 0, day 2, day 4 and day 7. Data are presented as mean ± SD (n=15). Plants were cultured in growth medium and exposed to different activity concentrations of IR from a 90Sr source. Dose rate values: 0, 0.82, 7.69 and 72.67 µGy•h-1. Within week statistical significance is represented by * (significance levels: p-value < 0.05 (*), p-value < 0.01 (**), p-value < 0.001 (***), p-value < 0.0001 (****); two-way ANOVA). µGy = microgray, h= hour. [file Image2.tif]

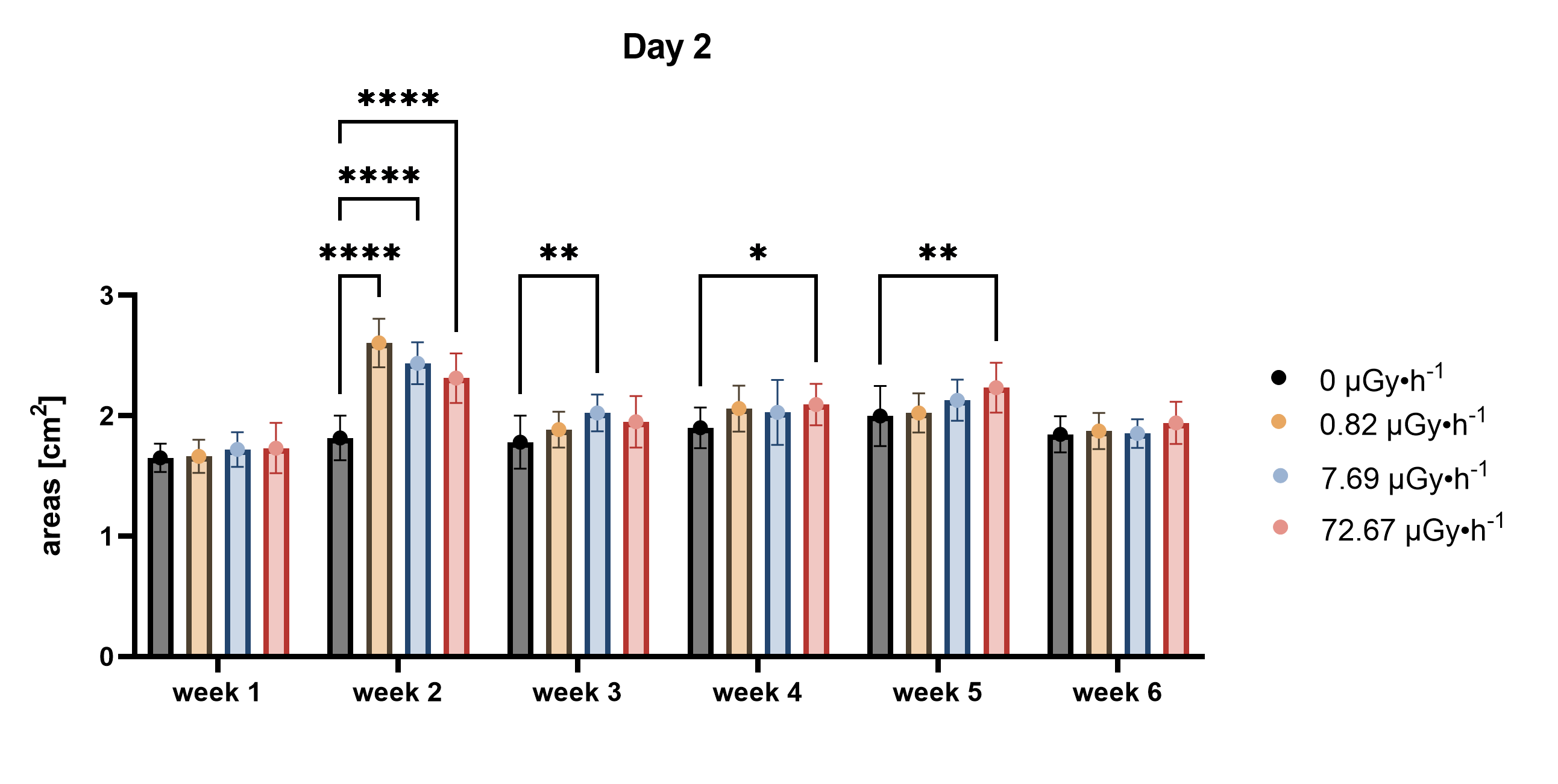

Supplement: Supplementary Figure 3 — Absolute values frond number. Overviews of frond number changes at day 0, day 2, day 4 and day 7. Data are presented as mean ± SD (n=15). Plants were cultured in growth medium and exposed to different activity concentrations of IR from a 90Sr source. Dose rate values: 0, 0.82, 7.69 and 72.67 µGy•h-1. Within week statistical significance is represented by * (significance levels: p-value < 0.05 (*), p-value < 0.01 (**), p-value < 0.001 (***), p-value < 0.0001 (****); two-way ANOVA). µGy = microgray, h= hour. [file Image3.tif]

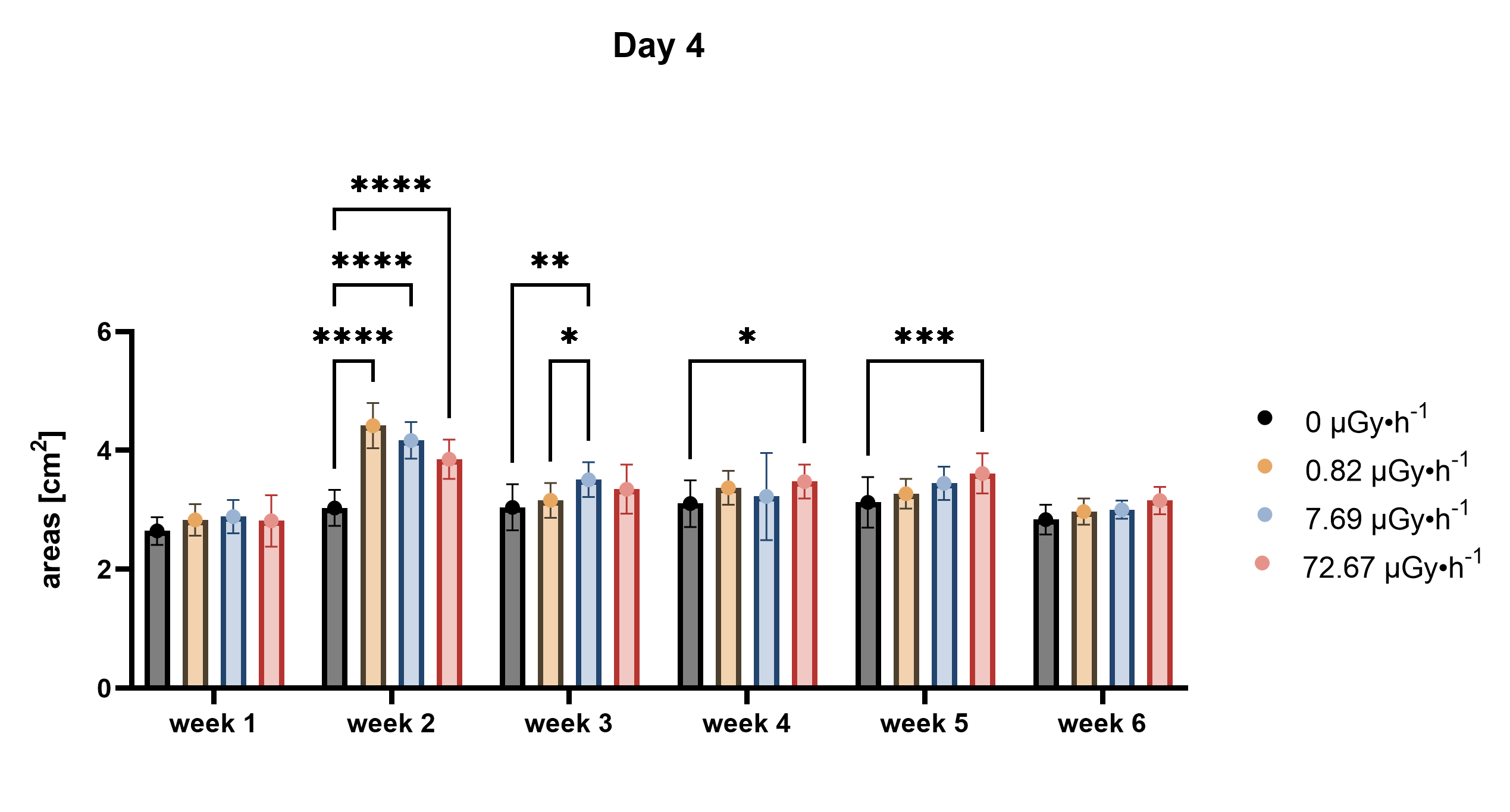

Supplement: Supplementary Figure 4 — Telomere length. Relative telomere length across weeks. Data are presented as mean ± SD (n=3). Plants were cultured in growth medium and exposed to different activity concentrations of IR from a 90Sr source: 0, 0.82, 7.69 and 72.67 µGy •h-1. Within week statistical significance is represented by * (significance levels: p-value < 0.05 (*), p-value < 0.01 (**), p-value < 0.001 (***), p-value < 0.0001 (****); two-way ANOVA). [file Image4.tif]

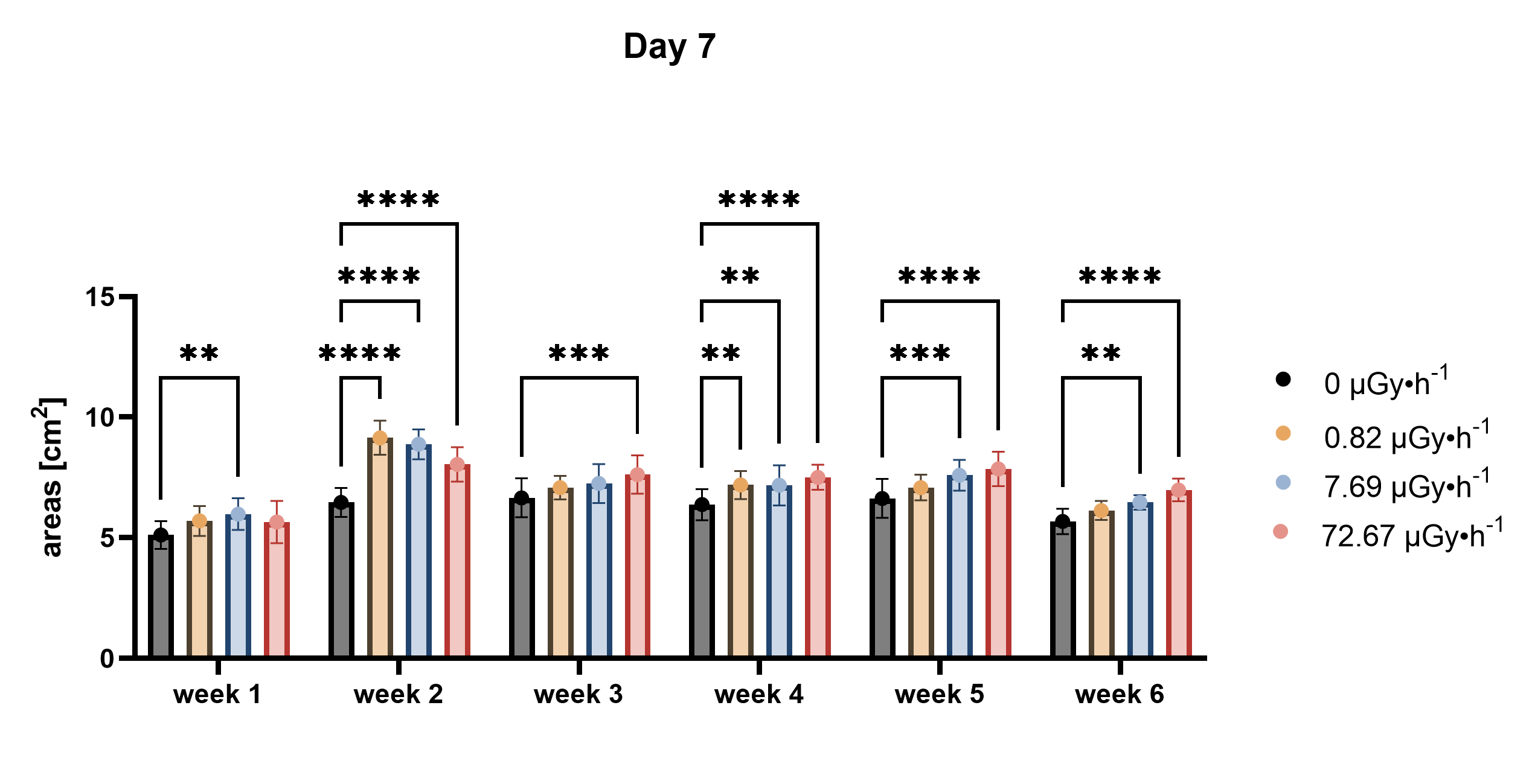

Supplement: Supplementary Figure 5 — ETR dynamics. (A-F) Electron transport rate of weeks 1–6 plotted against PAR irradiance values. Data are presented as mean ± SD (n=3). Plants were cultured in growth medium and exposed to different activity concentrations of IR from a 90Sr source. Dose rate values: 0 (control), 0.82 (β1), 7.69 (β2) and 72.67 (β3) µGy•h-1. Within week statistical significance is represented by the appropriate dose rate symbol (e.g. β3) coupled with an asterisk (significance levels: p-value < 0.05 (*), p-value < 0.01 (**), p-value < 0.001 (***), p-value < 0.0001 (****); two-way ANOVA). [file Image5.tif]

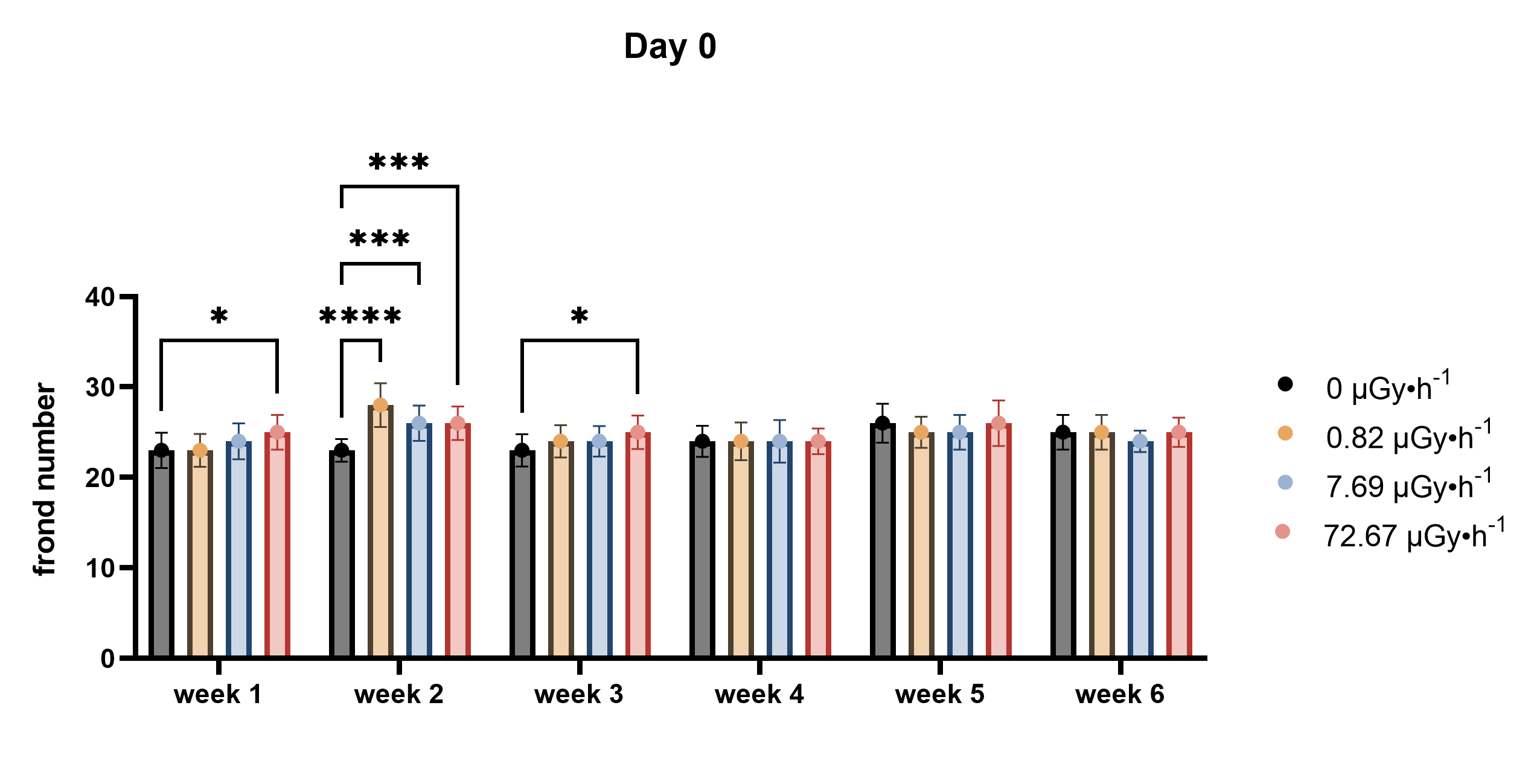

Supplement: Supplementary Figure 6 — Chlorophyll a to chlorophyll b ratio. Chlorophyll a to chlorophyll b ratio fluctuations. Data are presented as mean ± SD (n=3). Plants were cultured in growth medium and exposed to different activity concentrations of IR from a 90Sr source: 0, 0.82, 7.69 and 72.67 µGy •h-1. Within week statistical significance is represented by * (p-value < 0.05; two-way ANOVA). µGy = microgray, h= hour. [file Image6.tif]

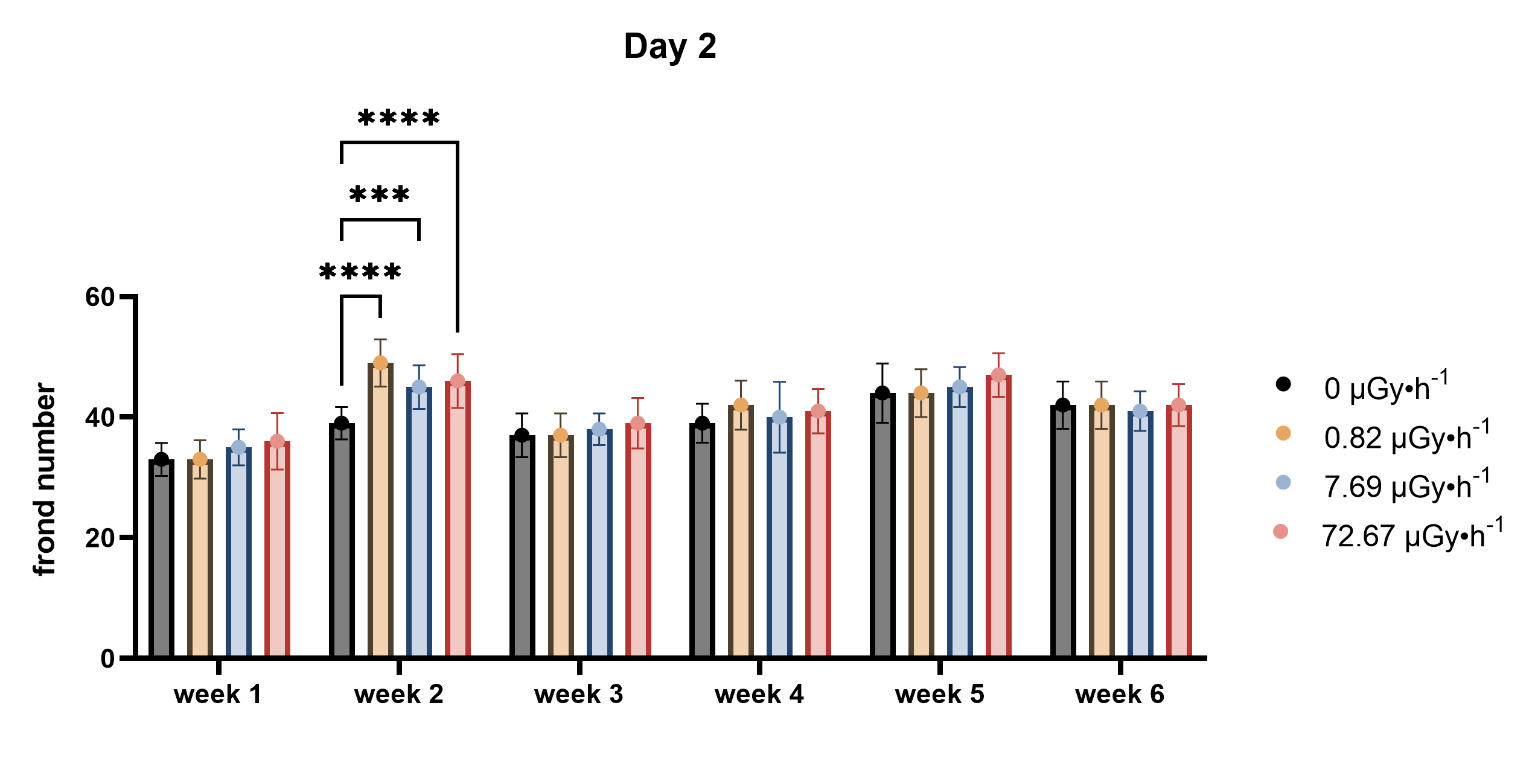

Supplement: Supplementary Figure 7 — Glutathione measurements. Overviews glutathione levels in samples from week 1, week 2 and week 6. Data are presented as mean ± SD (n=4). Plants were cultured in growth medium and exposed to different activity concentrations of IR from a 90Sr source. Dose rate values: 0, 0.82, 7.69 and 72.67 µGy•h-1. (A) Total glutathione content; (B) Reduced glutathione content; (C) Oxidized glutathione content; (D) Percentage of oxidized glutathione. Statistical significance is represented by * (significance levels: (p-value < 0.05 (*), p-value < 0.01 (**), p-value < 0.001 (***), p-value < 0.0001 (****); two-way ANOVA). µGy = microgray, h= hour. [file Image7.tif]

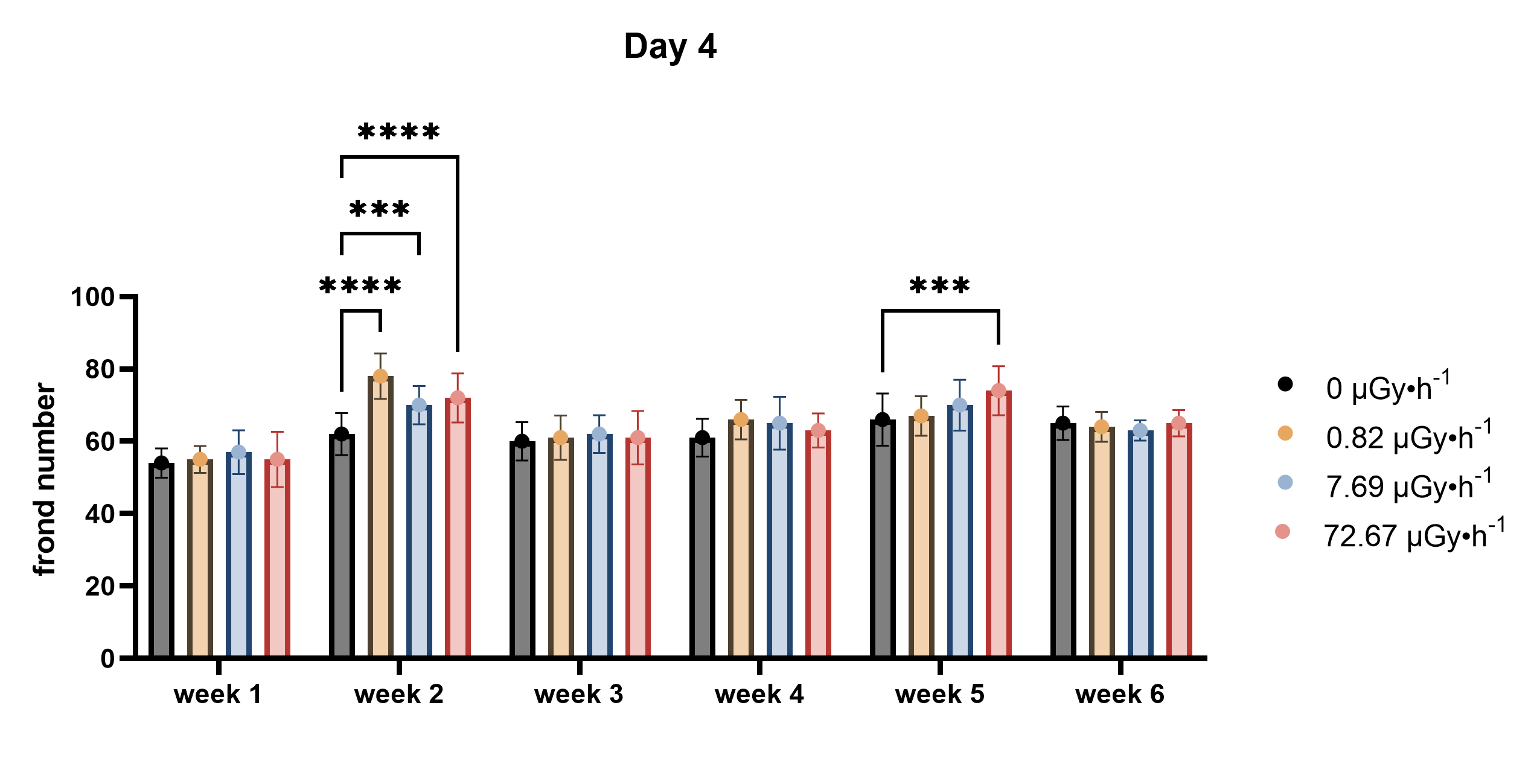

Supplement: Supplementary Figure 8 — Comprehensive stability of housekeeping genes. Graph showing the comprehensive stability ranking of reference genes (TUBB5, CYP, MAP2K1, BSL2, and SBT3.3) based on the geometric mean of their ranking values. Stability assessment was performed using RefFinder, a web-based tool that integrates geNorm, NormFinder, BestKeeper, and the comparative ΔCt method to assign weighted rankings to each gene. Lower geomean values indicate higher expression stability. TUBB5 = Tubulin beta-5 chain; CYP71A25 = Cytochrome P45071A25; MAP2K1 = Mitogen-activated protein kinase kinase 1; BSL2= Serine/threonine-protein phosphatase; SBT3.3 = Subtilisin-like protease. [file Image8.tif]

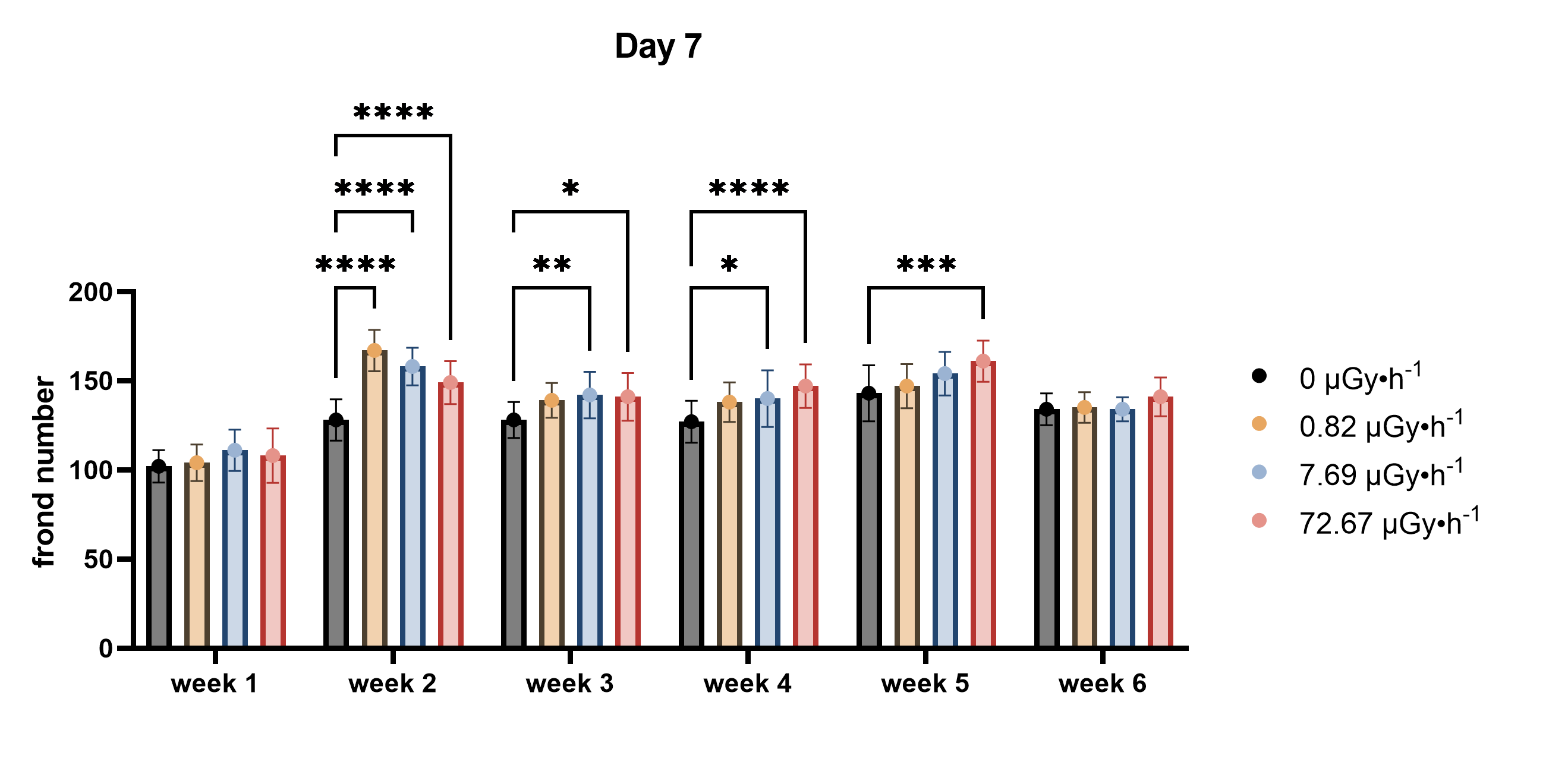

Supplement: Supplementary Figure 9 — Agarose gel electrophoresis of PCR products for genes of interest. PCR products of genes employed in the RT-qPCR analysis were separated on a 2% agarose gel stained with ethidium bromide and visualized under UV light. Lane A: GeneRuler Low Range DNA Ladder (Cat. No. SM1193, ThermoScientific). Lanes 1–10 correspond to the following genes: 1. DNA methyltransferase 1 (MET1). 2. Chromomethylase 3 (CMT3). 3. Repressor of silencing 1 (ROS1). 4. Sirtuin 1 (SRT1). 5. Glutamate-cysteine ligase (GSH1). 6. Glutathione synthetase 2 variant 1 (GSH2.1). 7. Glutathione synthetase 2 variant 2 (GSH2.2). 8. Glutathione reductase (GR). 9. Telomere reverse transcriptase (TERT). 10. Tubulin beta-5 chain (TUBB5). All genes show single bands at the expected sizes, confirming the specificity of the amplified PCR fragments. [file Image9.tif]

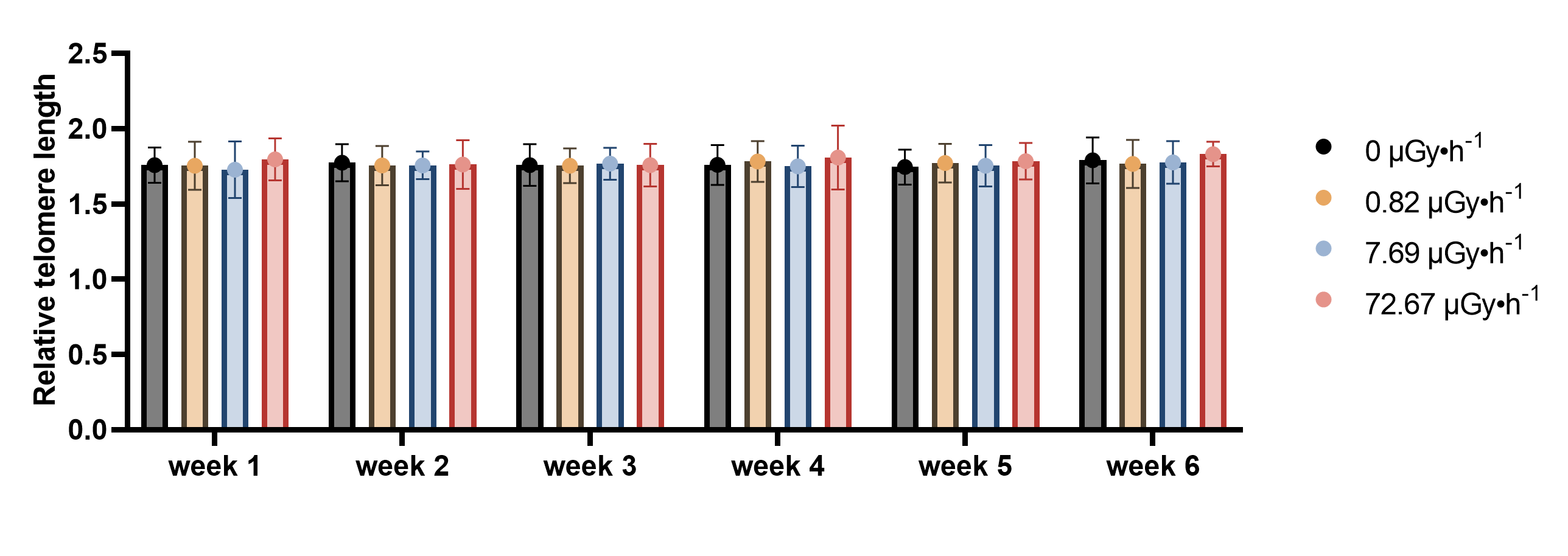

Supplement: Supplementary file 10 [file Image10.tif]

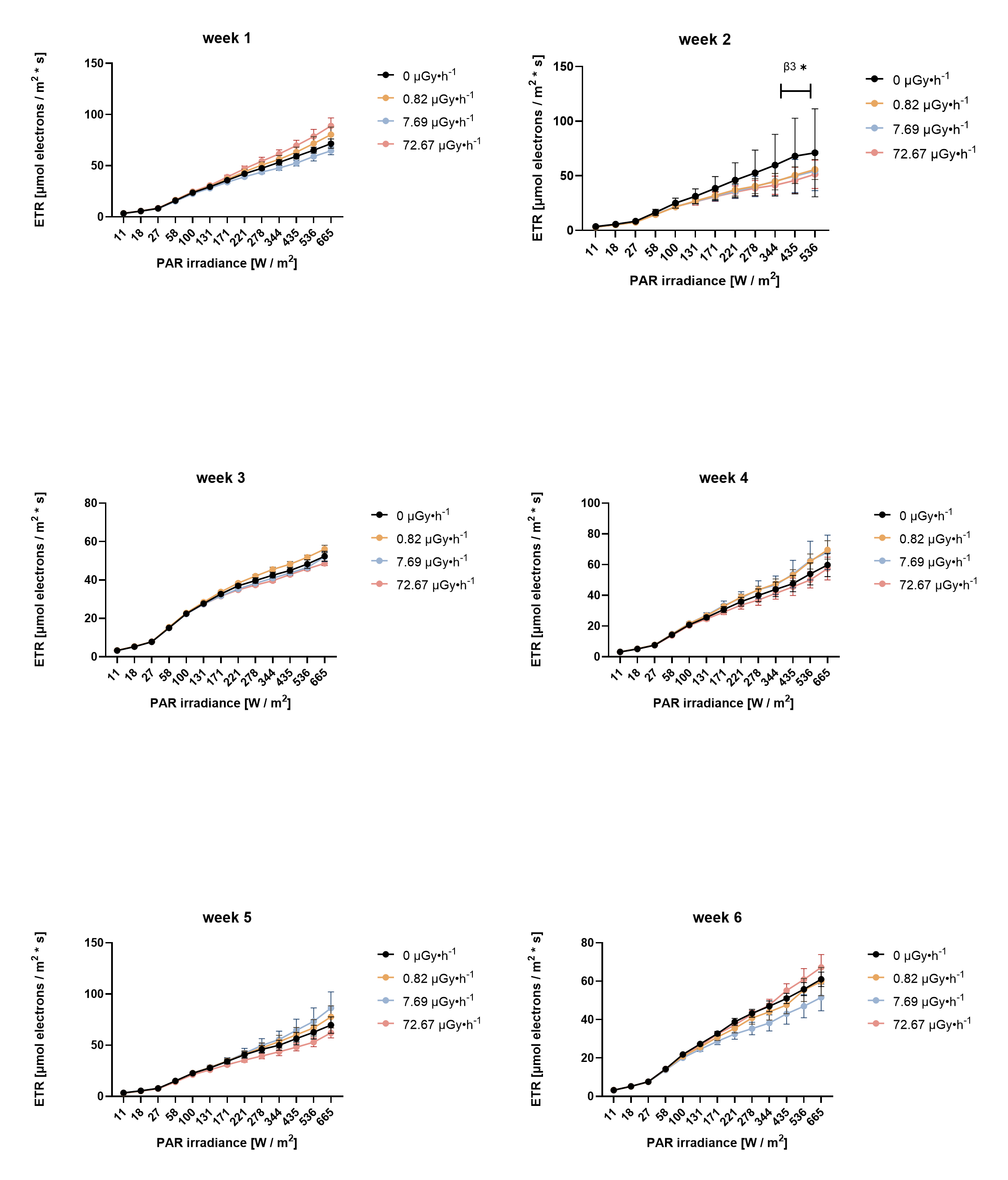

Supplement: Supplementary file 11 [file Image11.tif]

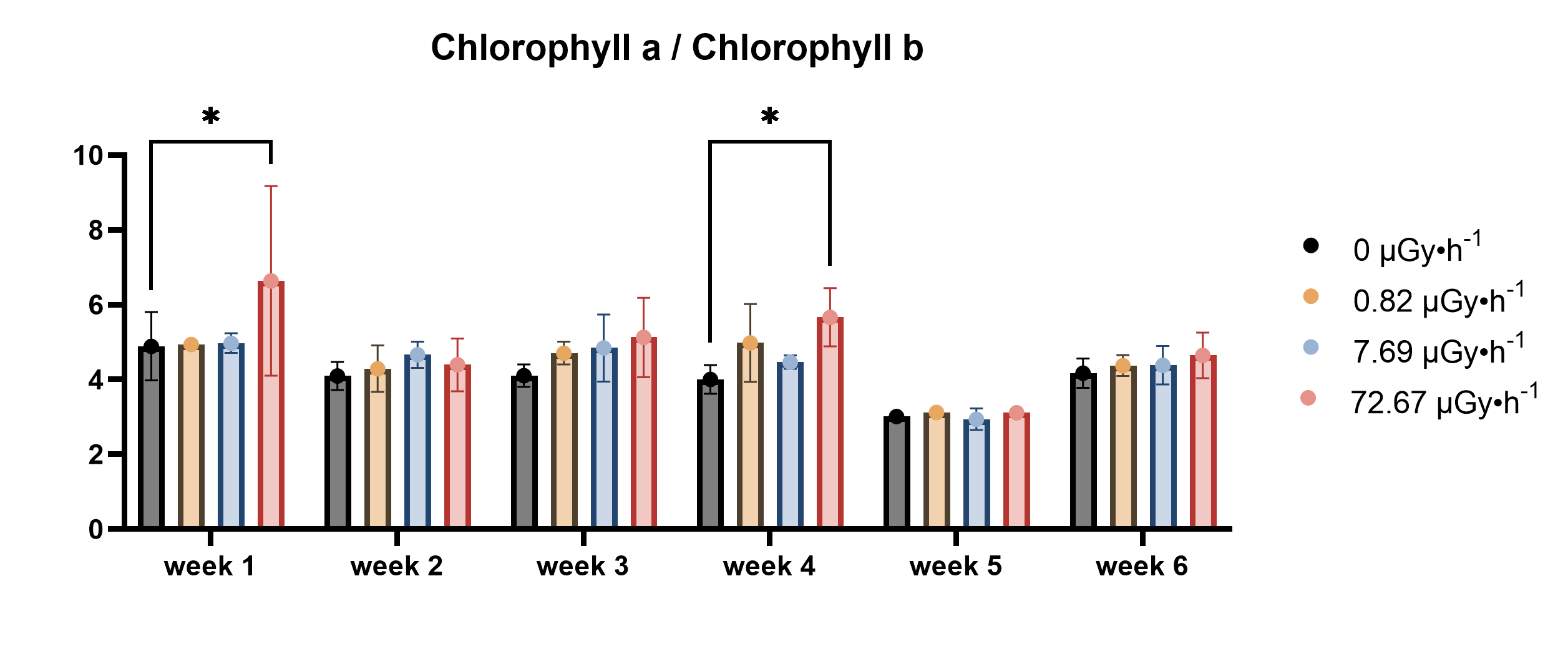

Supplement: Supplementary file 12 [file Image12.tif]

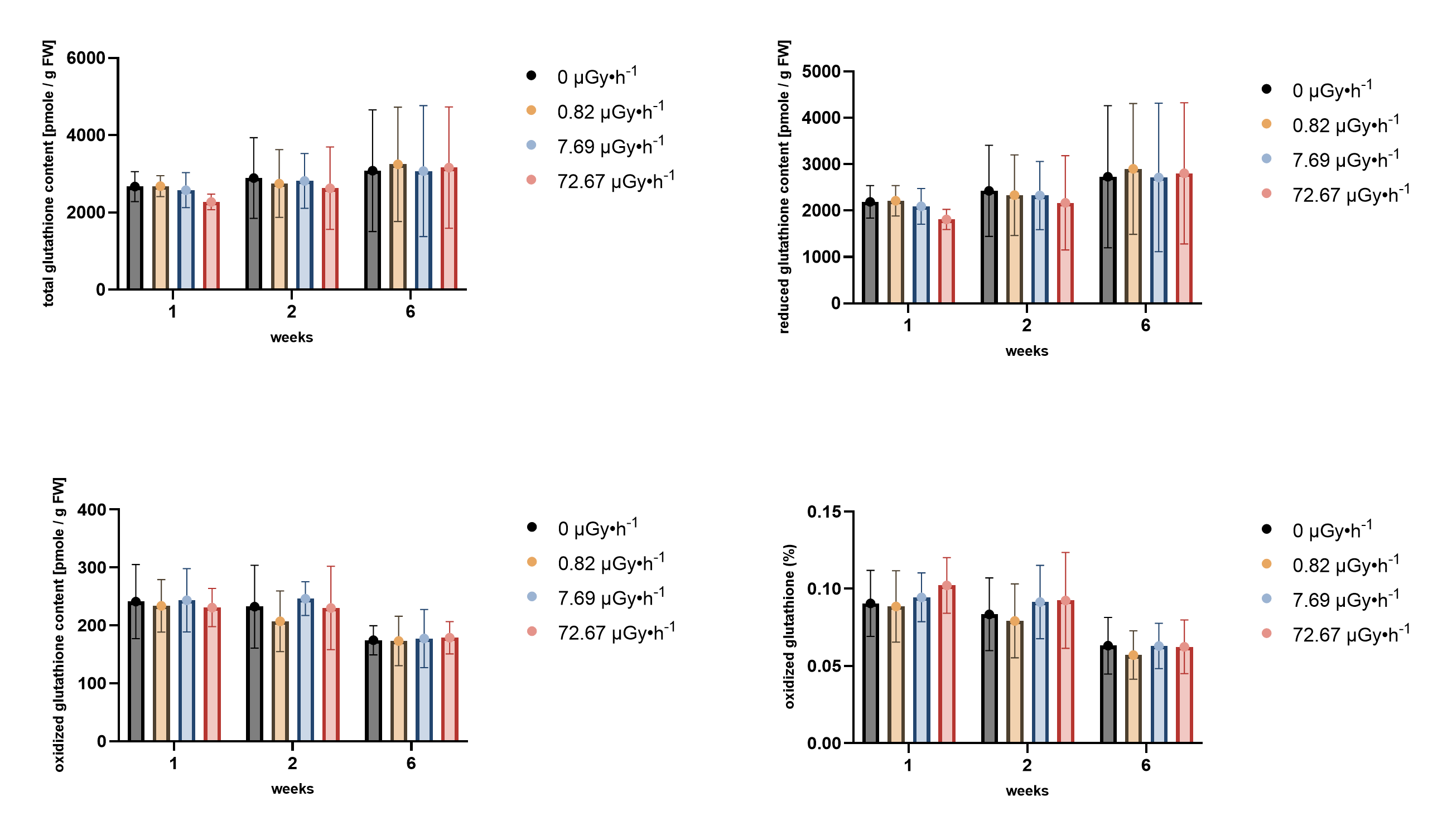

Supplement: Supplementary file 13 [file Image13.tif]

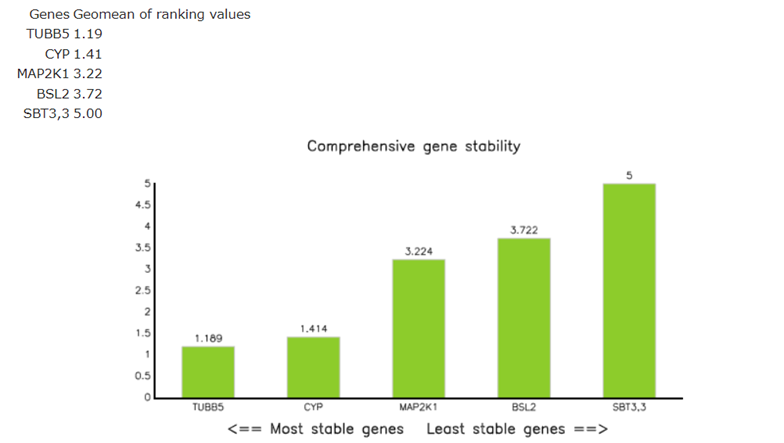

Supplement: Supplementary file 14 [file Image14.png]

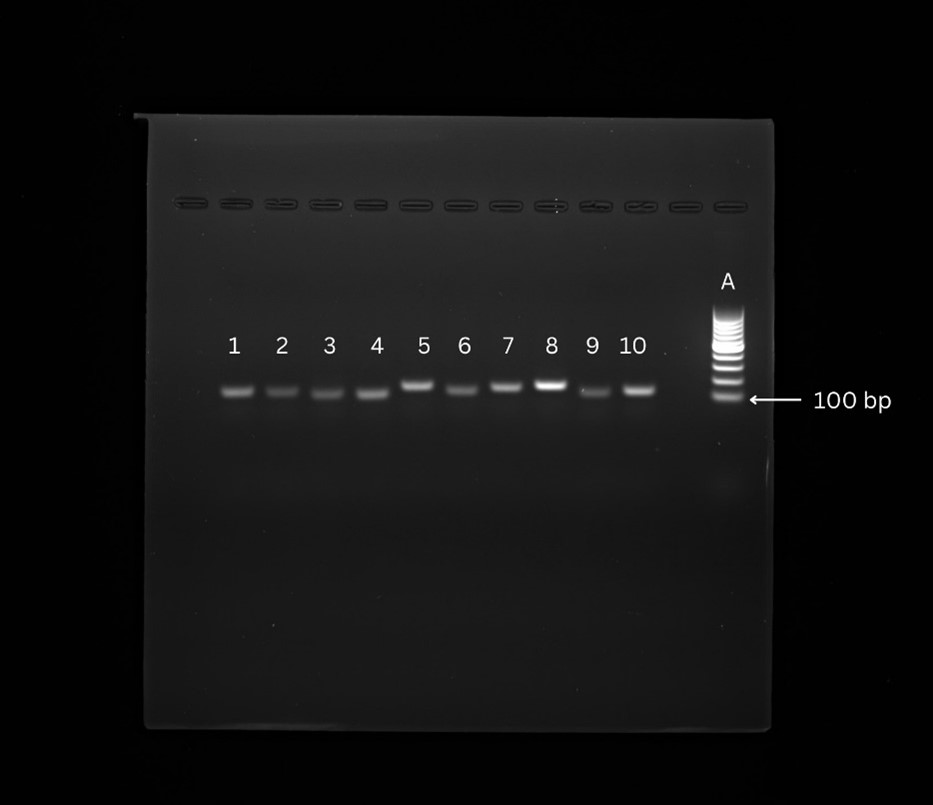

Supplement: Supplementary file 15 [file Image15.jpeg]
